# Evidence of natural reproduction of Atlantic sturgeon in the Connecticut River from unlikely sources

**DOI:** 10.1371/journal.pone.0175085

**Published:** 2017-04-07

**Authors:** Tom Savoy, Lorraine Maceda, Nirmal K. Roy, Doug Peterson, Isaac Wirgin

**Affiliations:** 1 Marine Fisheries Division, Connecticut Department of Energy and Environmental Protection, Old Lyme, Connecticut, United States of America; 2 Department of Environmental Medicine, New York University School of Medicine, Tuxedo, New York, United States of America; 3 Warnell School of Forestry and Natural Resources, University of Georgia, Athens, Georgia, United States of America; SOUTHWEST UNIVERSITY, CHINA

## Abstract

Atlantic Sturgeon is listed under the U.S. Endangered Species Act as five Distinct Population Segments (DPS). The “endangered” New York Bight (NYB) DPS is thought to only harbor two populations; one in the Hudson River and a second smaller one in the Delaware River. Historically, the Connecticut River probably supported a spawning population of Atlantic Sturgeon that was believed extirpated many decades ago. In 2014, we successfully collected pre-migratory juvenile specimens from the lower Connecticut River which were subjected to mitochondrial DNA (mtDNA) control region sequence and microsatellite analyses to determine their genetic relatedness to other populations coastwide. Haplotype and allelic frequencies differed significantly between the Connecticut River collection and all other populations coastwide. Sibship analyses of the microsatellite data indicated that the Connecticut River collection was comprised of a small number of families that were likely the offspring of a limited number of breeders. This was supported by analysis of effective population size (*Ne*) and number of breeders (*Nb*). STRUCTURE analysis suggested that there were 11 genetic clusters among the coastwide collections and that from the Connecticut River was distinct from those in all other rivers. This was supported by UPGMA analyses of the microsatellite data. In AMOVA analyses, among region variation was maximized, and among population within regions variation minimized when the Connecticut River collection was separate from the other two populations in the NYB DPS indicating the dissimilarity between the Connecticut River collection and the other two populations in the NYB DPS. Use of mixed stock analysis indicated that the Connecticut River juvenile collection was comprised of specimens primarily of South Atlantic and Chesapeake Bay DPS origins. The most parsimonious explanation for these results is that the Connecticut River hosted successful natural reproduction in 2013 and that its offspring were descendants of a small number of colonizers from populations south of the NYB DPS, most notably the South Atlantic DPS. Our results run contrary to the belief that re-colonizers of extirpated populations primarily originate in proximal populations.

## Introduction

Atlantic sturgeon *Acipenser oxyrinchus* is a large, long-lived, anadromous species that is widely distributed along the Atlantic coast of North America [1]. Spawning populations are found in most major river systems extending from St. Lawrence River, Quebec, to the Satilla River, Georgia [2]. Atlantic Sturgeon have a complex life history with considerable variation in growth, age of maturity and maximum longevity. Historically, there were at least 25–30 spawning populations of Atlantic Sturgeon coastwide [3], but that number has dwindled in recent decades to 15–20 populations [4, 5].

At one time, Atlantic Sturgeon spawning runs supported large riverine fisheries in the U.S. These fisheries primarily targeted spawning adults, particularly in the Delaware River [6] which had annual landings in the late 1890s of about three million pounds and a female population size of approximately180,000 [6]. By the beginning of the 20^th^ century, overexploitation had caused most riverine fisheries and populations to collapse to less than 10% of their historic highs [7]. In the mid and late 20^th^ century, smaller fisheries were re-established throughout the Atlantic coast, but these also collapsed within a few years [8]. All harvest within the U.S. was prohibited by a federal coastwide moratorium in 1998. In Canada, limited fisheries are still ongoing in the St. Lawrence River and the Saint John River, New Brunswick [9].

**Fig 1:**
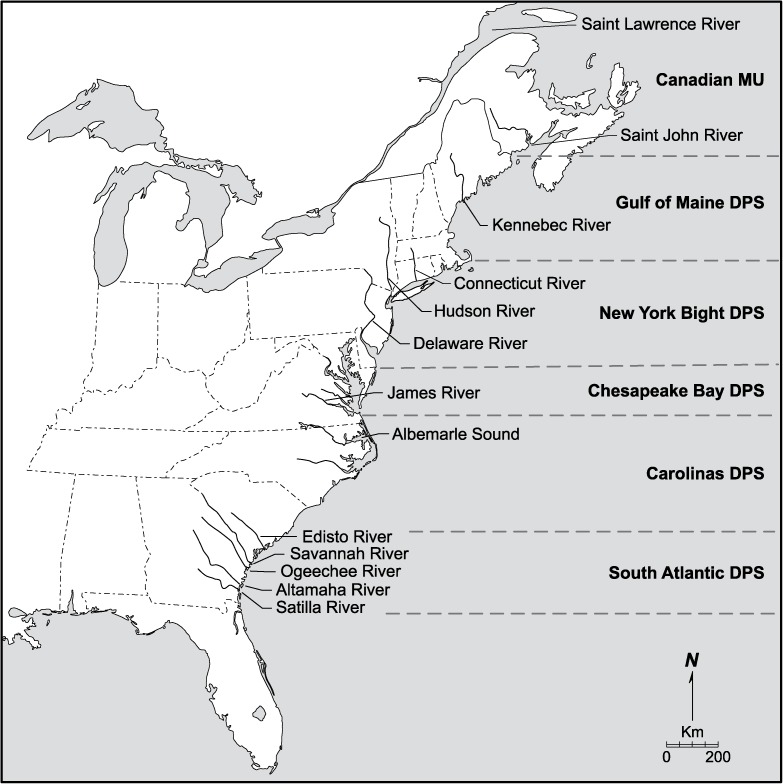
Map depicting the 12 spawning rivers where specimens were collected for this study and the demarcation of the five Distinct Population Segments (DPS) and Canadian Management Unit (MU) under which Atlantic sturgeon are managed under the U.S. Endangered Species Act and the Species at Risk Act (SARA) in Canada, respectively. Reprinted from (5) under a CC BY license, with permission from John Wiley & Sons, Inc., original copyright 2015.

A perceived failure of populations to rapidly rebuild following imposition of the U.S. moratorium resulted in a federal listing of Atlantic Sturgeon under the U.S. Endangered Species Act (ESA) in 2012. Under this listing, the National Marine Fisheries Service established five Distinct Population Segments (DPS) (Fig 1) based on regional differences in genetic structuring among populations.[10, 11]. Four of the DPS were designated as “endangered,” including that in the New York Bight (NYB), and the fifth in the Gulf of Maine was listed as “threatened.” Within Canada, the species was designated as “threatened” by the Committee on the Status of Endangered Wildlife in Canada and is being considered for more protective status through the Species at Risk Act.

Recent studies suggest that the NYB DPS currently contains one of the most robust populations remaining within U.S. waters. It includes estuarine and marine waters from Chatham, Massachusetts in Cape Cod to the Delaware-Maryland border and is known to support two naturally spawning populations in the Hudson River and a second considerably smaller one in the Delaware River. Threats that may impede stabilization and rebuilding of populations in the NYB DPS include bycatch in coastal commercial fisheries [5], habitat degradation [10, 11], vessel strikes [12], compromised water quality [13], chemical pollution [14], and construction of dams that impede access to historic spawning habitats [10].

The unusual life history characteristics of Atlantic Sturgeon are important in framing its genetic population structure. Atlantic sturgeon is anadromous with spawning occurring in natal rivers above the fall line over gravel, rubble, rocky substrate [1]. Juveniles are resident within natal rivers until 2–6 years of age; the exact age of this river residency phase is population dependent [1, 7, 15]. After leaving their natal rivers as subadults, they form coastal aggregations of mixed population and DPS origin [5, 16–18]. Within the marine environment, subadults and adults make long seasonal movements [19, 20] over prolonged periods through unknown migratory corridors [21, 22]. Migrants seasonally visit non-natal estuaries and coastal areas until returning to spawn in their natal rivers once sexually mature [15, 18]. There are latitudinal differences in age at first reproduction, with initial spawning for females ranging between 7–19 years in South Carolina rivers to 27–28 years in the St. Lawrence River [23]. After spawning, adults exit their natal estuaries, weeks to months after spawning, and resume their coastal movements. Timing between spawning events is also variable between males and females and may range from 1 to 5 years per individual. Males may spawn annually or every second year, whereas female spawning is thought to be more intermittent typically occurring at 3–5 year intervals [7]. The protracted and variable spawning intervals have confounded many previous attempts to estimate spawning population size in most river systems although annual run estimates have now been obtained for a limited number of populations [24–26].

Many genetic studies indicate that homing fidelity of Atlantic Sturgeon to natal rivers is strong. As a result, differentiation among spawning populations is significant based on frequencies of microsatellite DNA alleles [5, 27] and mitochondrial DNA (mtDNA) control region haplotypes [28–30]. The baseline genetic data that was used to quantify the genetic relatedness among Atlantic Sturgeon populations used in DPS designations was derived from spawning adults, young life-stages, or river-resident juveniles. These genetic differences among populations served as a foundation upon which the five DPS were distinguished. Furthermore, these genetic differences have allowed for the accurate identification of population and DPS origin of aggregations and individual subadults and adults captured in coastal waters [5, 16, 17] and non-natal estuaries [18] using mixed stock analysis and individual based assignment testing.

The Connecticut River has been known for several decades to host aggregations of subadult (> 50 cm and < 130 cm TL) Atlantic Sturgeon [18, 31]. In the 1800s and very early 20^th^ century, the Connecticut River probably supported successful natural reproduction of Atlantic Sturgeon as evidenced by a limited fishery for adults centered at Cromwell, CT [32] at river kilometer (rkm) 72.5 prior to the construction of several main stem dams, commercial fishing activity, and water quality problems [4]. Since 1988, low numbers of subadult Atlantic Sturgeon have been routinely collected by the Connecticut Department of Energy and Environmental Protection (CT DEEP) in fisheries surveys in the lower Connecticut River [31]. Annual presence of sturgeon in the lower reaches of the river led to speculation that a remnant stock persisted, but despite years of effort neither adults nor juvenile life-stages were encountered. However, Waldman et al. [18] believing that all subadults in the lower Connecticut River were seasonal migrants from elsewhere conducted mtDNA and microsatellite DNA analyses of specimens collected there from 1989 to 2011. They concluded that these subadults (mean TL = 937 mm) were primarily of Hudson River origin (65–70%), but also that some were spawned in all five U.S. DPS and in rivers as distant as those in the South Atlantic DPS.

Natural reproduction in Atlantic Sturgeon populations is typically confirmed by documenting the presence of mature adults at spawning locales at spawning time [33] or by the presence of young life-stages (eggs and larvae) [34] or pre-migratory juveniles [15, 35]. Although neither spawning adults nor young life-stages have been observed in the Connecticut River for almost 100 hundred years, the carcass of a 2.13 M female specimen with immature eggs was observed 17 rkm from the river’s mouth in spring 2014 [36] suggesting that natural reproduction may have resumed in the river.

We had two objectives in the present study; 1) to determine if natural reproduction of Atlantic sturgeon has persisted or recently resumed in the Connecticut River, and 2) if so, determine the genetic relatedness of juveniles collected there to other populations coastwide, most importantly to the two known populations in the NYB DPS. Our working hypothesis was that a small naturally reproducing population of Atlantic Sturgeon has persisted undetected in the Connecticut River and that its population would exhibit genetic characteristics distinct from all populations coastwide, but most similar to those in proximal populations in the NYB DPS. This hypothesized genetic result would provide strong evidence for the long-term persistence of a naturally reproducing population in the Connecticut River. A second alternate hypothesis was that a newly found cohort would be descendants of migrants from the Hudson River, the most proximal and largest population coastwide. A third less likely hypothesis was that a newly found cohort would be offspring of migrants from other more distant populations outside of the NYB DPS. To test these hypotheses, we characterized newly found specimens from the Connecticut River at 11 microsatellite loci and the mitochondrial DNA (mtDNA) control region and compared their allelic and haplotype frequencies to those in 10 (microsatellites) and 11 (mtDNA) other coastwide populations, respectively.

## Materials and methods

### Study site

The Connecticut River is the largest river in New England, flowing 660 km from its source on the Canadian-Vermont border to Saybrook, Connecticut on Long Island Sound. The river’s watershed encompasses 29,163 square kilometers in four U.S. states and its 555 cubic meter/second discharge produces 70% of the freshwater flow into Long Island Sound. Historically, the main stem Connecticut River hosted 13 dams, the most downstream of which, the Enfield Dam, was constructed in 1829 at rkm 0. Because the dam was relatively low head, it was thought to be an obstruction to anadromous fishes only during low flows. A center section was removed to facilitate fish passage in 1933 and additional major breaches were noted by 1976.

### Sturgeon sampling

A skiff trawl (9.7 m x 7.0 m dimensions, a 2.0 cm mesh codend, and a 0.5 cm mesh codend liner) was fished in the lower Connecticut River between river kilometers (rkm) 6–18 in May and June, 2014. The trawl was fished against the predominant river flow for 8–15 minutes at approximately 1.5 knots groundspeed at water depths ranging from 2.4–9.7 m. Tow duration and distance covered was a function of known bottom topography, obstructions, environmental conditions and vessel traffic.

Gill nets (2.3 m high by 100 m long, single mesh size per net of 2.5 to 10.1 cm stretched mesh) were fished from July through October, 2014. Nets were weighted to sample bottom waters and anchored parallel to the predominant current and were set for a maximum of 2 h around slack water. Typically, four nets were fished per outing in water depths varying from 7.6 to 15.2 m from rkm 6–18.

All sturgeon were placed into a 350-L live well with ‘StressCoat’ and flow through water. Fish were individually placed into a water-filled examination box for measurement and tagging. Specimens were measured for total length (TL) and fork length, inter-orbital distance, mouth width, and scanned for previous tags and general health. All untagged sturgeon received a Passive Integrated Transponder (PIT) tag that was injected into the left side below the dorsal fin and a 1 cm^2^ piece of anal fin was removed for genetic analyses and stored in 95% ethanol. Ages of juvenile Atlantic sturgeon were determined based on modal distributions in length frequency histograms as described previously (2).

Specimens in reference collections from other spawning populations used for comparative purposes were either juveniles < 50 cm TL or adults > 130 cm TL because it is assumed that these two life-stages are natal to the rivers in which they are collected. Specimens > 50 cm and < 130 cm TL are considered subadults and because of their wide-ranging migratory behavior are not necessarily natal to the rivers in which they are collected. Mitochondrial DNA results for reference samples (Table 1) were originally reported in [2] except for 421 new samples that supplemented those earlier collections. These were from the Saint John River (n = 59 adults), Hudson River (n = 56 juveniles), Delaware River (n = 8 juveniles), James River (n = 58 adults), Albemarle Sounds (n = 28 juveniles), Edisto River (n = 70 juveniles), Savannah River (n = 57 juveniles), Ogeechee River (n = 45 juveniles), and the Altamaha River (n = 40 juveniles). Additionally, data from 9 Kennebec River specimens reported in (2) were removed in the current mtDNA data set because of uncertainty regarding their sizes. All microsatellite DNA reference collection data that we report here (Table 2) are new to this study and may be different from that in (5). All new specimens from the South Atlantic DPS reported in this study were collected under University of Georgia Animal Use and Care Permit No. A2013 01-012-Y3-A1 issued to DP.

**Table 1:** Locations where Atlantic Sturgeon collections characterized for mtDNA control region haplotypes were made from 12 rivers, their latitude-longitude coordinates, sample size (*N*), sampling dates, and total length range (mean total length).

Populations	*Lat-Long Coordinates*	*N*	Sampling Dates	Total Length Range (cm) (mean) or Maturity State
**St. Lawrence River**	(49.175809, -67.254181)	46	Aug 1992	All subadult males
**Saint John River**	(45.260751, -66.066799)	76	July-Aug 1992; July-Aug 1993	All spawning adults
		59	July 2014	162.6–248.9 (199.7)
**Kennebec River**	(45.260751, -66.066799)	19	June-July 1980	155–208 (170.3)^1^
		11	June 2010	152–196 (171.3)
		31	June-Aug 2011	132.8–197.4 (171.7)
		3	Aug-Nov 2011	15–46.2 (25.4)
**Connecticut River**	(41.274895, -72.335186)	45	May-Oct 2014	22.5–71.0 (53.7)
**Hudson River**	(40.703379, 74.027166)	91	June 1990–1994	All spawning adults
		26	June 1996	172–201 (185)
		25	June-July 1997	170–218.4 (183.6)^2^
		30	July 2006	156–242 (192.4)
		41	June 2009	165.1–210.8 (190.8)
		50	June 2010	170.2–222.3 (197.7)
		30	Mar-Apr 2011	43.2–54 (49.8)
		35	Mar-Apr 2013	41.1–52.8 (46.2)
**Delaware River**	(38.873625, -75.020828)	60	Sept-Nov 2009	22.0–35.7 (29.3)
		47	Sept-Nov 2011	23.5–36.3 (28.9)
**James River**	(36.983554, -76.303310)	72	Apr 1997-Feb 1998	26.0–49.5 (45.7)
		59	July-Sept 2014	93.0–211 (160.0)^1^
**Albemarle Sound**	(35.938644, -76.724138)	40	May-Sept 1998	28.6–48.5 (38.8)
		2	Aug-Sept 1997	134–142.2 (138.1)
		5	July 1997	39–40.9 (39.7)
		46	Dec 2006-Mar 2014	27–54.1 (41.6)
**Edisto River**	(32.481220, -80.357780)	51	Apr-Oct 1996	27.7–50 (39.9)
		21	May-Oct 1998	116–233.7 (164.5)
		21	June-July 2001–2003	26–49.6 (39.2)
		2	June-July 2003	183.2–193.1 (188.2)
		47	May-Sept 2005	32.6–48.5 (42.4)
**Savannah River**	(32.019929, -80.880489)	3	Oct-Nov 1997	146.4–155.6 (152)
		17	Oct-Dec 1997	38–48.8 (43.8)
		16	Apr-Oct 1998	29.5–50.0 (43.2)
		16	Aug-Nov 1999	32.2–49.0 (42.5)
		30	Mar-Nov 2000	30.6–49.7 (41.6)
		3	July 2005	39.5–47.0 (43.2)
		45	May-June 2013	31.6–44.4 (39.1)
**Ogeechee River**	(31.841608, -81.069660)	3	June 2000	28.2–39.4 (32.7)
		32	June-Dec 2003	15.3–49.2 (30.9)
		12	Mar-Oct 2004	21.3–43.3 (31.5)
		26	June 2007-Aug 2009	19.9–38.3 (27.6)
		45	July-Aug 2014	22.7–31.0 (26.0)
**Altamaha River**	(31.317192, -81.299686)	9	Aug-Sept 1993	35.5–50 (44)
		31	2004	161.3–217.9 (181.4)
		50	June-July 2005	31.9–40.4 (37.9)
		50	Apr-May 2005	139.3–209.8 (171.3)
		40	July-Aug. 2011	32.7–49.0 (38.6)

^1^ Fork length

^2^ Total length data on 12 of 25 specimens

**Table 2:** Locations where Atlantic Sturgeon characterized for microsatellite DNA genotypes were collected from 11 rivers, sample size (N), sampling date, and total length range (mean total length).

Populations	*N*	Sampling Date	Total Length Range (cm) (mean) or Maturity State
**Saint John River**	66	July-Aug 1992; Aug 1993	All Spawning adults
	59	July 2014	162.6–248.9 (199.7)
**Kennebec River**	43	June 2010-Aug 2011	133–197.4 (171.6)
**Connecticut River**	45	May-Oct 2014	22.5–71.0 (53.7)
**Hudson River**	30	Mar-Apr 2011	43.2–54 (49.8)
	35	Mar-Apr 2013	41.1–52.8 (46.2)
	46	Apr-May 2014	28.7–48.9 (43.9)
**Delaware River**	59	Sept-Nov 2009	22.0–36.7 (29.3)
	49	Sept-Nov 2011	23.5–36.3 (28.9)
**James River**	58	Apr 1997-Feb 1998	26.0–49.5 (45.7)
	58	July-Sept 2014	93.0–211 (160.0)^1^
**Albemarle Sound**	41	May-Sept 1998	28.6–48.5 (38.8)
	31	Dec 2006-Jan 2011	27.0–49.9 (40.3)
	17	Jan 2013-Mar 2014	31.5–49.4 (43.9)
	2	Nov 2013; Feb 2014	132–155 (143.5)
**Edisto River**	53	Apr-0ct 1996	27.7–50 (39.9)
	52	May-Sept 2005	32.6–48.5 (42.4)
**Savannah River**	50	May-June 2013	31.6–44.7 (39.1)
	50	May 2014	27.4–47.9 (37.0)
**Ogeechee River**	26	June 2007-Aug 2009	19.9–52.0 (28.6)
	45	July-Aug 2014	22.7–31.0 (26.0)
**Altamaha River**	49	June-July 2005	31.9–40.4 (37.9)
	40	July-Aug 2011	32.7–48.1 (38.6)

^1^ Fork length

### DNA isolations

Fin clips were washed with phosphate-buffered saline, and incubated in cetyltrimethyl ammonium bromide (C-Tab) buffer [37] and digested at 65^o^ C with proteinase K (Roche Diagnostics, Indianapolis, IN). DNAs were purified by phenol-chloroform extractions, alcohol precipitated, air dried and resuspended in Tris-EDTA buffer as described in [38]. Concentrations and purities of DNAs were evaluated using a Nanodrop ND-1000 Spectrophotometer (NanoDrop Technologies, Wilmington, DE). DNA concentrations were adjusted to 50 ng/μl for standardization of subsequent analyses.

### Mitochondrial DNA control region sequence analysis

A 560 bp portion of the mtDNA control region was amplified with derived Atlantic Sturgeon-specific primers S1 (5'- ACATTAAACTATTCTCTGGC- 3') and G1 (5'- GAATGATATACTGTTCTACC- 3') [39]. The same primers were used to sequence most of the 560 bp amplicon. We report here data on only 205 bp of the amplicon to allow for comparison of haplotypes in Connecticut River specimens to previously characterized reference collections from other rivers [2, 24, 28–30].

Polymerase chain reactions (PCRs) were in 50 μl volumes that contained 50 ng of template DNA, 5 μl of 10 x Roche Applied Science (Indianapolis, IN) reaction buffer, 0.25 μl of each dNTP (25 mM stocks) (GE Healthcare, Piscataway, NJ), 0.07 μl of S1 primer (0.1 μM stock), 0.05 μl of G1 primer (0.1 μM stock) (Integrated DNA Technologies, Coralville, IA), 1 unit of Taq DNA Polymerase (Roche Applied Science) and 43.9 μl of _dd_H_2_0. Amplification conditions were 94^0^ C for 5 min followed by 40 cycles at 94^0^ C for 45 s, 56^0^ C for 45 s, 72^0^ C for 60 s, followed by a final extension at 72^°^C for 10 min in MJ Research PTC-100^TM^ thermal cyclers. Amplicons were purified with QIAquick PCR Purification kits (Qiagen, Valencia, CA).

Purified PCR products were Dye-Terminator Cycle Sequenced as recommended in GenomeLab Methods Development kits by the manufacturer (Beckman Coulter, Inc., Fullerton, CA). Sequencing conditions were 30 cycles at 96^0^ C for 20 s, 50^0^ C for 20 s, and 60^0^ C for 240 s. Sequencing products were ethanol precipitated, re-suspended in 40 μl of Beckman Coulter CEQ Sample Loading Buffer, loaded into a Beckman Coulter CEQ^TM^ 8000 automated capillary-based DNA sequencer, run using the standard long fast read method (LFR-1), and analyzed with the Sequence Analysis Module of the CEQ^TM^ 8000 Genetic Analysis System.

### Microsatellite analysis

Eleven microsatellite loci were scored that were previously shown to be effective in distinguishing reference collections from spawning populations of Atlantic Sturgeon [5, 17, 27]. These included LS19, LS39, LS54, LS68 [40], Aox23, AoxD45 [41], and Aox44, AoxD165, AoxD170, AoxD188, AoxD241 [42].

Microsatellite PCRs were in 12.5 μl volumes that contained 50 ng of template DNA, 1.25 μl of 10 x Roche Applied Science (Indianapolis, IN) or 10 x KlenTaq1 reaction buffer (AB Bioscience, LLC, St. Louis, MO), 0.1 μl of each dNTP (25 mM stocks) (GE Healthcare), 0.5 μl of both labeled (Sigma Aldrich, Woodlands, TX) and unlabeled primers (Integrated DNA Technologies) (1.0 μM stock), 0.05 μl (1 unit) of Taq DNA Polymerase (Roche Applied Science) (LS19, LS39, AoxD170) or 0.025 μl of KlenTaq (25 units/μl) (all other loci) and _dd_H_2_0 to volume. Initial denaturation was at 95^0^ C for 5 min and 55 cycles were at 95^0^ C for 15 s, 60^0^ C (except AoX45 at 62^°^C, Aox23 at 64^0^ C, and LS19, LS39, and AoxD170 at 50^0^ C) for 15 s, 72^0^ C for 30 s, and 72^0^ C for 7 min.

Microsatellite genotypes were determined using the Beckman Coulter sequencer. Individual PCR reactions were multi-pooled, diluted up to 1:3 with Sample Loading Solution (Beckman Coulter), 0.5–2.0 μl of reactions were loaded onto 96 well plates along with 0.5 μl of CEQ DNA Size Standard-400 and 40 μl of Sample Loading Solution (Beckman Coulter), and run with the FRAG 1 program (Beckman Coulter).

### Statistical analysis

#### Mitochondrial DNA data analysis

Individual specimens were assigned mtDNA haplotypes based on discrete combinations of nucleotides at polymorphic sites. Mitochondrial DNA sequence diversity within reference and the Connecticut River collections was assessed in Arlequin v. 3.5.2.2 [43] by enumerating their number of haplotypes, haplotype diversity [44], and nucleotide diversity [45].

Population structure was evaluated using the Φ_*ST*_ approach implemented in Arlequin. Φ_*ST*_ estimates consider both the frequencies of haplotypes in collections and the genetic distances among haplotypes. Values of Φ_*ST*_ were used to estimate effective number of female migrants in the equation *N*_*e*_*m*_*f*_ = ((1/Φ_*ST*_)-1)/2) [46].

Hypotheses of population structure as suggested by Bonferroni-corrected Φ_*ST*_ analysis were further evaluated using analysis of molecular variance (AMOVA) [47] implemented in Arlequin. The optimal groupings tested by AMOVA were those in which variation among regional groupings was maximized and variation among populations within groupings minimized. Significance of all hierarchical AMOVA analyses was assessed through 9999 permutations. We used AMOVA specifically to empirically test if genetic data supported the placement of the Connecticut River juvenile collection within the NY Bight DPS or not.

#### Microsatellite data analysis

Microsatellite data was initially examined using MicroChecker v/2.2.3 [48] to identify the presence of null alleles, scoring errors, and/or large allele drop-out. Exact tests in GENEPOP (version 4.2) [49, 50] were used to test the genotypes at each locus and in each collection for their conformance to Hardy Weinberg equilibrium (HWE). Linkage disequilibrium (LD) was tested for all pairs of loci in each collection using contingency tables also implemented in GENEPOP. All tests of HWE and LD used the default Markov chain parameters in GENEPOP. Significance levels for HWE and LD tests were adjusted using sequential Bonferroni correction [51]. Microsatellite allelic diversity at all loci and in all collections was quantified in GenAlEx v. 6.503 [52], FSTAT v. 2.9.3 [53], and HP Rare 1.0 [54]; measures presented include proportion of polymorphic loci (*P*), number of alleles per locus (*N*_A_), rarified allelic richness (*A*_*R*_), allelic richness (*A*), expected heterozygosity (*H*_E_) and observed heterozygosity (*H*_*O*_) and inbreeding coefficient (*F*_*IS*_).

The significance of allelic differentiation among these collections and between the 2014 Connecticut River juvenile collection and Connecticut River subadults reported in Waldman et al. [18] was originally evaluated using exact G tests implemented in GENEPOP using default Markov chain parameters. Further evaluation of population structuring among collections using Wright’s [55] *F*_ST_ was implemented in FSTAT [53] using the **θ** estimator of Weir and Cockerham [56]. For highly variable microsatellite markers such as those used in this study, *F*_ST_ may not the best measure of genetic differentiation among collections because the maximum value of *F*_ST_ = 1 cannot be obtained even when collections have completely non-overlapping sets of alleles [57]. Therefore, we also used GenAlEx to calculate *F*’_ST_, which scales *F*_ST_ values based on observed allelic diversity so that *F’*_ST_ always equals 1 when no alleles are shared. Both indices of genetic differentiation are useful because if both indicate a similar pattern, one can be more confident in the population structure observed [58]. Significance of all pairwise *F*_ST_ and *F*’_ST_ comparisons were assessed through 9999 permutations.

To evaluate the possibility that the Connecticut River juvenile collection represented a recently established cohort from a limited number of founders, we determined if it consisted of a small number of families, each with many full-sibling dyads, compared to juvenile collections from other rivers in the NYB DPS. We compared the number of full sibling dyads within the Connecticut River to the number in juvenile collections from the other two populations within the NY Bight DPS, the Hudson River (one of the larger populations coastwide) and the Delaware River (one of the smallest populations coastwide) using the program COLONY v. 2.0 [59, 60].

For the Hudson River, we determined the number of full sibling dyads in juvenile collections made in 2011, 2012, and 2014. For the Delaware River, we determined the number of full-sibling dyads for juvenile collections made in 2009 and 2011. Samples were analyzed with the assumptions of no per locus genotyping error (although we did not empirically test this), male and female polygamy, no inbreeding, medium run length with the full likelihood analysis method, and high likelihood without assignment of individuals as candidate males or females as these data were not available to use. Although our inference of family relationships is weakened by the absence of age, sex, and relationship information, and the assumption of polygamy for both sexes, COLONY is predicted to be more accurate than pairwise estimates of relationships [59]. As a result of the large number of full-sibling dyads and small number of families (n = 11) identified within the Connecticut River collection compared to those within the Hudson or Delaware rivers, only one individual from each Connecticut River family was represented in subsequent analysis of evolutionary relationships among populations described below. However, the deletion of many full-sibs left us with quite small sample sizes for subsequent analysis described below.

Clustering among individuals within and among collections was analyzed using STRUCTURE v. 2.3.4 [61]. STRUCTURE uses a Bayesian model to infer the number of genetic clusters (*K*) among collections, assign individuals to individual clusters, and identify individuals of mixed ancestry. STRUCTURE determines the number of genetic clusters among collections by optimizing Hardy Weinberg equilibrium and linkage equilibrium within clusters. We used the admixture model in STRUCTURE using sampling locations as a prior with allele frequencies correlated. We used burn-in lengths of 10,000 and run lengths of 100,000 and ten replicates were done for each run of *K* = 1–12. The best value of *K* was determined from values of lnP(D) [61] and Δ*K* [62] using STRUCTURE HARVESTER [63]. Output files from STRUCTURE were illustrated using Clumpak [64].

The evolutionary relationship among Atlantic Sturgeon collections based on the microsatellite data was visualized through the construction of UPGMA trees in POPTREE2 [65]. To further describe among population differentiation based on the microsatellite data, we used the hierarchical AMOVA approach as described above.

As a result of its small number of families determined with the COLONY, we compared the effective population size (*N*_*e*_) and effective number of breeders *(N*_*b*_*)* of the Connecticut River 2014 juvenile cohort to those for the Hudson River and Delaware River based on 2011, 2013, 2014 juvenile collections and 2009 and 2011 juvenile collections, respectively. The Hudson River and Delaware River collections were chosen for comparison because they are also within the New York Bight DPS and because robust numbers of juveniles were available from each. We used the bias-corrected single-sample linkage disequilibrium method [66] implemented in NeEstimator v. 2.01 [67] to estimate contemporary *N*_*e*_ and *N*_*b*_ based on the microsatellite data.

Finally, mixed stock analysis (MSA) and individual based assignment (IBA) testing based on the microsatellite and mtDNA data implemented in ONCOR [68] were used to estimate the population and DPS origin of specimens in the Connecticut River juvenile collection. ONCOR uses conditional maximum likelihood to estimate mixture proportions. Ninety-five % confidence intervals to MSA mixture estimates were determined using the bootstrapping method of Rannala and Mountain [69]. IBA was used to assign individuals in the Connecticut River aggregation to the reference collection that would have the highest probability of producing the given genotype/haplotype in the mixed juvenile collection It should be noted that our analysis of a combination of diploid and haploid mtDNA data violates an assumption of this Monte Carlo resampling method.

## Results

**Fig 2:**
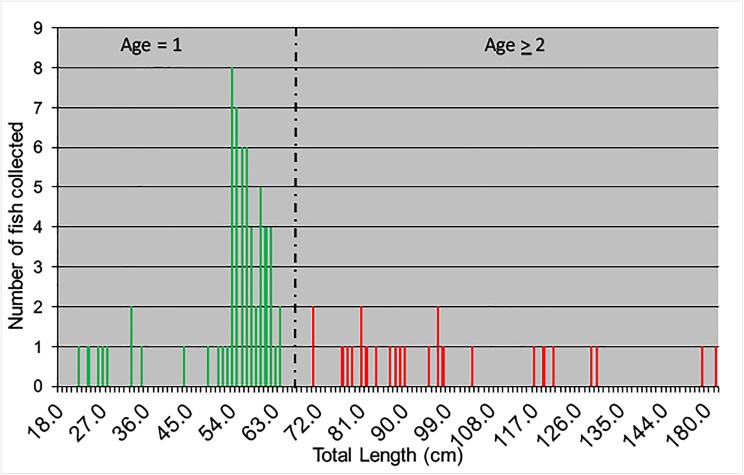
Length frequency histogram of the 2014 Connecticut River Atlantic Sturgeon collection. Green bars represent individual juvenile specimens from which 45 individuals were analyzed genetically. Red bars represent specimens that were not genetically analyzed because of their larger size.

In 2014, 64 juvenile Atlantic Sturgeon ranging from 22.5 to 71.0 cm TL were collected within the lower portion (rkm 6–18) of the Connecticut River from May through October during CT DEEP Marine Fisheries sturgeon research efforts (Fig 2). In total, the 45 smallest specimens (22.5 to 64.0 cm TL) were genetically analyzed. Twenty-three larger Atlantic Sturgeon were also collected and are depicted in the length frequency histogram (Fig 2), but were not analyzed genetically because they were likely older than the one-year old pre-migratory specimens that were the focus of our study.

Eight of the smallest Atlantic Sturgeon ranging from 22.5–33.0 cm TL were collected with skiff trawls from May 20, 2014 to June 25, 2014. Most were collected as a single fish per tow/day except for three that were caught on June 25, 2014. Fifty-six Atlantic Sturgeon were collected in gill net sets from July through October 2014. Three specimens were collected in July, one in early August, and the remaining 52 from September 22 until October 29, 2014. Not all juvenile sturgeon collected were genetically analyzed as several fish were recaptured and tissue samples were not taken from some others.

Recaptured fish served to document survival, relatively rapid growth, and assisted in determining the age of juvenile specimens. One Atlantic Sturgeon captured on June 12, 2014 increased from 27.0 cm TL to 58.5 cm TL by Oct. 6, 2014. A second recapture from first collection/tagging in September 2014 occurred in October 2014 with a similar rate of increase in length in 30 days.

Sequence analysis of the mtDNA control region revealed 35 haplotypes among 1,519 specimens from 12 collections of Atlantic Sturgeon spanning the species’ coastwide distribution (Table 3). The number of mtDNA haplotypes, haplotype diversity, and nucleotide diversity in the Connecticut River collection were often lower than in other collections except for those to its north in the Gulf of Maine DPS and Canada (Table 4) which were likely more impacted by the relatively recent effects of Pleistocene glaciation. For example, haplotype diversity in the Connecticut River collection was 0.246 compared to 0.550 and 0.541 in the Hudson River and Delaware River collections within the NYB DPS, respectively.

**Table 3:** Frequencies of mtDNA control region sequence haplotypes in 12 coastwide collections of Atlantic Sturgeon juvenile and adult specimens.

Populations												Haplotypes																								
	A	A1	A3	A5	A6	B	B1	B2	C	C1	C2	C3	C4	C5	C6	D	D2	D5	E	E2	E3	N	N1	N2	N3	O	P	P1	P5	P7	P8	P9	P10	P12	S1	Total
**St Lawrence**	46																																			46
**Saint John**	135																																			135
**Kennebec**	58	4				1							1																							64
**Connecticut**	1																		39									1		4						45
**Hudson**	90					201	4	4					10	15		4																				328
**Delaware**	7			28		67																	5													107
**James**	29					3			40				1			2	2		2			1	39				9									131
**Albemarle**	54					1										4	3		3				13				9								6	93
**Edisto**	79				1	1			18	2	9					17			6	1	1	3	2	1				1								142
**Savannah**	34		2			2			10		1	2			1	24		3	19	1		3	6	1		6	4		1	1	3	1	1	4		130
**Ogeechee**	8		5						8		1					69			1			1	1	2	3	19										118
**Altamaha**	67								32							10			70												1					180
**Total**	608	4	7	28	1	276	4	4	108	2	11	2	12	15	1	130	5	3	140	2	1	8	66	4	3	25	22	2	1	5	4	1	1	4	6	1519

**Table 4:** Indices of microsatellite allelic and mitochondrial DNA control region sequence haplotype diversity in 11 (microsatellites) and 12 (mtDNA) collections of Atlantic Sturgeon. Collection locale, microsatellite DNA results [sample size (*n*), proportion of polymorphic loci (*P*), number of alleles (*N*_*A*_), allelic richness (*A*), observed heterozygosity (*H*_O_), and expected heterozygosity (*H*_E_)], inbreeding coefficient (*F*_*IS*_), and mitochondrial DNA results [sample size (*n*), number of haplotypes (*n*_H_), haplotype diversity (*h*), nucleotide diversity (π), and mean number of pairwise differences.

	Microsatellite DNA							Mitochondrial DNA				
Population	*n*	*P*	*N*_*A*_	*A*	*H*_O_	*H*_E_	*F*_*IS*_	*n*	*n*_H_	*h*	π	Pairwise Differences
**St. Lawrence**	nd	nd	nd	nd	nd	nd	nd	46	1	0.000	0.0000	0.000
**Saint John**	125	1.00	8.46	6.84	0.600	0.613	0.023	135	1	0.000	0.0000	0.000
**Kennebec**	43	90.9	8.18	7.96	0.654	0.637	-0.015	64	4	0.292	0.0014	0.279
**Connecticut**	47	90.9	5.64	4.69	0.804	0.593	-0.372	45	4	0.246	0.0070	1.427
**Hudson**	111	1.00	9.73	8.24	0.680	0.667	-0.014	328	7	0.550	0.0063	1.282
**Delaware**	108	1.00	8.73	7.48	0.658	0.650	-0.007	107	4	0.541	0.0052	1.055
**James**	116	1.00	10.5	8.83	0.676	0.683	0.012	128	10	0.775	0.0105	2.059
**Albemarle**	91	1.00	9.27	8.05	0.685	0.690	0.012	93	8	0.633	0.0114	2.323
**Edisto**	105	1.00	8.09	6.99	0.657	0.647	-0.011	142	13	0.664	0.0092	1.869
**Savannah**	100	1.00	9.73	8.30	0.673	0.688	0.026	130	22	0.856	0.0167	3.380
**Ogeechee**	71	1.00	9.18	8.23	0.639	0.668	0.050	118	11	0.641	0.0144	2.929
**Altamaha**	89	1.00	8.18	7.47	0.672	0.681	0.018	180	5	0.679	0.0075	1.524

This, and previous studies, revealed highly significant differences in haplotype frequencies among all collections except for among the most northern collections from the St. Lawrence and Saint John rivers (Table 5). The Connecticut River juvenile collection was significantly different from all others coastwide, including those from the Hudson and Delaware rivers, the other two populations within the NY Bight DPS (Table 5). Most interesting was the identity of the haplotypes within the Connecticut River collection (Table 3). The A haplotype is common coastwide and in 84% of Kennebec River and 27% of Hudson River specimens, the two populations in closest proximity to the north and south, respectively, of the Connecticut River (Table 3). However, the A haplotype was only present in one specimen (2%) from the Connecticut River. The E haplotype, by far the predominant haplotype in the Connecticut River collection, was observed in 39 of 45 (87%) of its specimens. The other two haplotypes in the Connecticut River collection were P1 (2%) and P7 (8%). Other than the Connecticut River, the E, P1, and P7 haplotypes were only observed in specimens from populations in the Carolinas and SA DPS. The E haplotype was particularly common in the Altamaha River collection where it was detected in 38.6% of specimens. P1 and P7 were only observed in single specimens, each from the Edisto and Savannah rivers, respectively. Based on the mtDNA data, female mediated gene flow between the Connecticut River collection and all others was low, < 1, except for the Altamaha River where it was estimated at 2.38 (Table 5). Importantly, female mediated gene flow between the Connecticut River and the Hudson River and Connecticut River, was low, 0.54 and 0.46, respectively.

**Table 5:** Matrix of ΦST comparisons (below diagonal) and effective number of female migrants estimates (above diagonal) among 12 collections of juvenile and adult Atlantic Sturgeon based on mitochondrial DNA control region haplotypes using *Nemf* in the equation *Nemf* = ((1/ΦST)-1)/2).

	St. Lawrence	Saint John	Kennebec	Connecticut	Hudson	Delaware	James	Albemarle	Edisto	Savannah	Ogeechee	Altamaha
**St. Lawrence**		Infinity	14.200	0.915	1.401	0.837	1.036	4.468	3.251	1.446	0.685	2.565
**Saint John**	***0*.*000***		10.753	0.206	1.130	0.514	0.670	2.313	2.071	0.787	0.381	1.642
**Kennebec**	***0*.*013***	0.047		0.454	1.549	0.945	1.039	4.780	3.367	1.374	0.661	2.600
**Connecticut**	0.547	0.708	0.524		0.537	0.457	0.701	0.870	1.092	1.133	0.599	2.379
**Hudson**	0.263	0.307	0.244	0.482		5.050	0.770	1.476	1.381	0.840	0.519	1.182
**Delaware**	0.374	0.493	0.346	0.523	0.068		0.758	1.331	1.290	1.023	0.627	1.074
**James**	0.326	0.427	0.325	0.417	0.394	0.397		2.556	3.384	7.435	1.779	1.731
**Albemarle**	0.101	0.178	0.095	0.365	0.253	0.273	0.164		11.096	3.534	1.358	4.723
**Edisto**	0.133	0.195	0.129	0.328	0.266	0.279	0.129	0.043		4.515	1.796	7.172
**Savannah**	0.257	0.389	0.267	0.306	0.373	0.328	0.063	0.124	0.100		6.780	2.285
**Ogeechee**	0.422	0.568	0.431	0.455	0.491	0.444	0.219	0.269	0.218	0.069		1.067
**Altamaha**	0.163	0.233	0.161	0.174	0.297	0.318	0.224	0.096	0.065	0.180	0.319	

All pairwise ΦST comparisons are statistically significant (*P* <0.001) except for those italicized in bold.

Microsatellite DNA analysis at 11 loci was completed on 1,006 specimens from 11 of the 12 collection sites that were analyzed for mtDNA haplotypes (St. Lawrence River omitted). Use of Microchecker found no evidence of genotyping errors due to large allele dropout or scoring of stutter peaks at any locus, however, null alleles were observed at 3 loci in 1–2 collections each. Since no single locus or population consistently departed from expectations, eliminating locus- and population-specific factors as causes for the deviations, all loci were retained for subsequent analyses.

We found no consistent evidence of Hardy-Weinberg or linkage disequilibria after Bonferroni correction in any population or at any pair of loci except for in the Connecticut River collection where 8 of 11 loci exhibited Hardy-Weinberg disequilibrium and 23 of 45 locus pairs displayed linkage disequilibrium. Consistent with the mtDNA results, several measures of allelic diversity were lower in the Connecticut River collection compared to others, including number of alleles/locus (5.64) and allelic richness (4.69) (Table 4). However, observed heterozygosity in the Connecticut River collection (0.804) was the highest coastwide.

Significantly different allelic frequencies were observed between the Connecticut River and all other collections coastwide. Using Exact G tests, all collections were significantly differentiated (*X*^*2*^ = infinity, *P* = Highly Significant) (data not shown) except between those from the James and Savannah rivers (*X*^*2*^ = 36.53, *P* = 0.027). Similarly, both *F*_*ST*_ and *F’*_*ST*_ analyses revealed significant differentiation between all collections coastwide (Table 6). For both *F’*_*ST*_ and *F*_*ST*_ analyses, the magnitude of the mean pairwise differentiation between the Connecticut River and all other coastwide collections (mean = *F’*_*ST*_ = 0.423 and *F*_*ST*_ = 0.161) was greater than the magnitude of differentiation among all other pairwise comparisons of collections. Of particular note was the extent of allelic differentiation between the Connecticut River and the two other collections within the NY Bight DPS. *F’*_*ST*_ and *F*_*ST*_ values between the Connecticut River and the proximal Hudson River collections were 0.497 and 0.186, respectively. Similarly, the *F’*_*ST*_ and *F*_*ST*_ values between the Connecticut River and the Delaware River collection were even greater at 0.547 and 0.291, respectively. In summary, both the mtDNA and microsatellite data suggested a closer genetic affinity of the Connecticut River collection to others in the SA DPS than to the two other populations within the NYB DPS.

**Table 6:** Matrix of pairwise *F’*_*ST*_ values above the diagonal and pairwise *F*_*ST*_ values below the diagonal based on microsatellite DNA analysis at 11 loci in 11 collections of Atlantic Sturgeon juveniles (< 50 cm TL) or adults (> 130 cm TL).

	Saint John	Kennebec	Connecticut	Hudson	Delaware	James	Albemarle	Edisto	Savannah	Ogeechee	Altamaha
**Saint John**		0.125	0.547	0.180	0.240	0.205	0.376	0.430	0.417	0.317	0.433
**Kennebec**	0.047		0.531	0.104	0.176	0.209	0.31	0.373	0.349	0.264	0.373
**Connecticut**	0.225	0.213		0.497	0.547	0.393	0.348	0.323	0.320	0.334	0.289
**Hudson**	0.065	0.036	0.186		0.077	0.179	0.280	0.362	0.338	0.274	0.379
**Delaware**	0.088	0.062	0.210	0.026		0.189	0.321	0.418	0.388	0.326	0.403
**James**	0.072	0.070	0.148	0.058	0.063		0.162	0.295	0.244	0.208	0.271
**Albemarle**	0.132	0.104	0.130	0.090	0.106	0.051		0.160	0.109	0.159	0.150
**Edisto**	0.159	0.133	0.133	0.124	0.146	0.099	0.054		0.093	0.219	0.084
**Savannah**	0.145	0.115	0.122	0.108	0.127	0.076	0.034	0.047		0.162	0.046
**Ogeechee**	0.114	0.091	0.129	0.108	0.110	0.067	0.052	0.075	0.052		0.201
**Altamaha**	0.153	0.125	0.113	0.123	0.134	0.148	0.047	0.028	0.014	0.065	

All pairwise *F’*_*ST*_ and *F*_*ST*_ values are statistically significant at *P* < 0.001.

COLONY analysis of family structure among the 45 juvenile specimens from the Connecticut River revealed that its collection was dominated by the presence of a large number of full-sibling dyads (Table 7). In total, the Connecticut River collection contained 704 out of a possible 1,128 full-sibling dyads. Only 8 (18%) of the Connecticut River specimens were not associated with a full-sibling dyad. In comparison, the 2009 and 2011 Delaware River juvenile cohorts harbored 10 and 25 full-sibling dyads respectively, with 90% and 80% of its individuals not associated with full-sibling dyads No full-sibling dyads were observed within three years of juvenile collections from the Hudson River.

**Table 7:** Comparison of relatedness of juvenile Atlantic Sturgeon collected in the Connecticut River in 2014, three year classes of juveniles from the Hudson River (2011, 2013, 2014) and two year classes of juveniles from the Delaware River (2009, 2011) as revealed by analysis of microsatellite genotypes in the COLONY program. Full sibling dyads refer to individuals that share the same two parents.

Population	Year Collected	N	Number Full Sibling Dyads	% Individuals w/out Full Siblings	Number Families
Connecticut River	2014	45	704	18%	11
Hudson River	2011	30	0	100%	30
Hudson River	2013	35	0	100%	35
Hudson River	2014	46	0	100%	46
Delaware River	2009	59	10	90%	51
Delaware River	2011	49	25	80%	39

Because of the extensive family structure in the Connecticut River juvenile cohort, we proceeded to compare its effective population size and number of breeders (*N*_*e*_ and N_*b*_) to other cohorts of juveniles within the NYB DPS including what is thought to be the largest population coastwide in the Hudson River and one of the smallest in the Delaware River (Table 8). *N*_*e*_ was estimated at 2.4 (2.1, 2.6) for the Connecticut River cohort compared to 26.9 (95% CI; 22.7, 32.2) and 34.8 (95% CI; 28.8, 42.9) for the two Delaware River cohorts and 261.8 (95% CI; 88.3-Infinite), 158.9 (95% CI; 83.3, 880.3), and 264.9 (95% CI; 127.2, 16,186) for the three Hudson River juvenile cohorts. In fact, the Connecticut River cohort exhibited by far the smallest *N*_*e*_ of any population coastwide that we have characterized (Waldman et al. unpublished data).

**Table 8:** Comparison of estimates of effective population sizes (*N*_*e*_) and effective number of breeders (*N*_*b*_) in collections of juvenile Atlantic Sturgeon from the Connecticut River in 2014, Hudson River in 2011, 2013 and 2014, and Delaware River in 2009 and 2011. *N*_*e*_ and *N*_*b*_ estimates were done in NeEstimator v.2. *N*_*e*_ estimates were made using the linkage disequilibrium method with 0.02 being the lowest allele frequency used. *N*_*b*_ estimates were made with the molecular co-ancestry method.

Juvenile Cohort	*N*_*e*_	95% CI	*N*_*b*_	95% CI
Connecticut River (2014)	2.4	2.1–2.6	3.5	2.5–6.4
Hudson River (2011)	261.8	88.3-Infinite	87.6	15.1-Infinite
Hudson River (2013)	158.9	83.3–880.3	22.7	11.0-Infinite
Hudson River (2014)	264.9	127.2–16,186	Infinite	19.2-Infinite
Delaware River (2009)	26.9	22.7–32.2	90.5	14.2-Infinite
Delaware River (2011)	34.8	28.8–42.9	22.1	11.4–1,886.7

**Fig 3:**
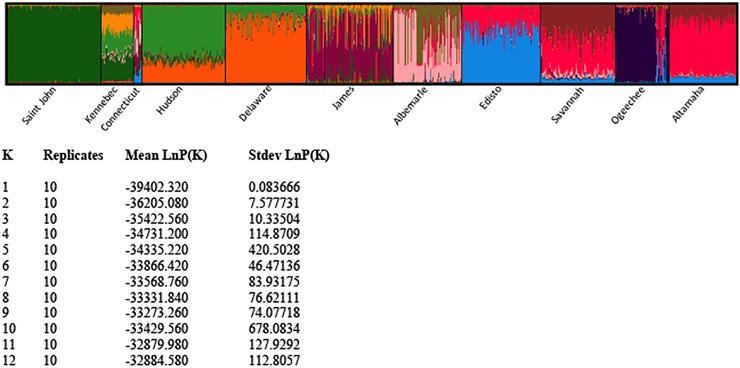
Bar plot and table of Ln Pr (X/K) value of results of STRUCTURE analysis (K = 11). The optimal delta K value was also 11. Results are based on microsatellite DNA analysis at 11 loci in 11 collections of Atlantic sturgeon. All collections were of juveniles (< 50 cm TL) or adults (> 130 cm TL) specimens except for that from the Connecticut River which included 5 juveniles between 50 and 56 cm TL.

STRUCTURE analysis offered the opportunity to define population units by iteratively sorting individual microsatellite genotypes into clusters to maximize the fit of the data to theoretical expectations derived from Hardy-Weinberg and linkage equilibria. Its use enabled us to infer the number of genetically homogenous clusters across all collections and allow assignment of individual specimens to designated genetic clusters. Because of the high number of full-sibling dyads and small number of families represented in the Connecticut River collection only one individual from each family (n = 11) was included in the analysis. We found 11 genetic clusters within our complete data set and these clusters usually corresponded with the collection locales of the specimens comprising them (Fig 3). Of particular importance for this study is that all specimens from the Connecticut River collection comprised a unique cluster that was distinct from the clusters representing the Hudson River and Delaware River collections in the NYB DPS.

**Fig 4:**
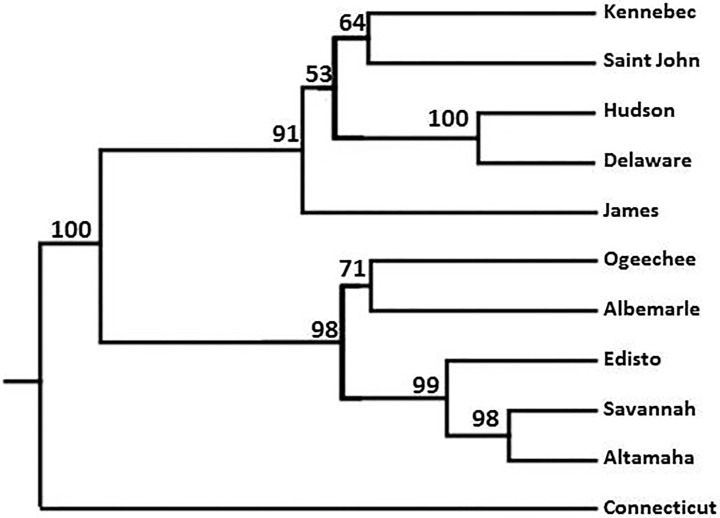
Evolutionary relationships among 11 collections of Atlantic Sturgeon juveniles and adults based on data from 11 microsatellite loci and constructed using the unweighted pair-group method with arithmetic averages (UPGMA) in POPTREE2

The significant genetic distance between the Connecticut River collection and the other two populations within the NYB DPS was reflected in phylogenetic analyses using UPGMA dendrograms developed microsatellite distance data (Fig 4). In the dendrogram, the node containing the Connecticut River collection was highly distinct from all others including those containing the Hudson River and Delaware River collections in the NYB DPS. As expected, the microsatellite dendrogram depicted two major branches; populations in the Chesapeake Bay DPS and north and populations in the Carolinas and SA DPS. The Connecticut River branch appeared basal to all other branches in the tree.

AMOVA analyses of the microsatellite and mtDNA data were used to evaluate several models of population structure suggested by the various statistical analyses discussed above (Table 9). The best model identified by AMOVA is that which maximizes variation among regions and minimizes variation among populations within regions. Our objective in this analysis was to determine whether the Connecticut River collection grouped best with the NYB DPS or outside of it. In AMOVA analysis of both the mtDNA and microsatellite data, the best model tested was that with 7 groupings that included the Canadian populations, the five U.S. DPS, and the Connecticut River. For both AMOVA analyses, including the Connecticut River collection within the NYB DPS reduced among region variation and increased variation among populations within regions.

**Table 9:** Analysis of Molecular Variance (AMOVA) of biologically relevant groupings among 11 (microsatellites) or 12 (mtDNA) collections of Atlantic sturgeon juveniles (<50 cm TL) and adults (>130 cm TL) based on mtDNA control region sequence and microsatellite analysis at 11 loci. Regional groupings tested include those identified as Distinct Population Segments (DPS) under the U.S. Endangered Species Act.

Groupings	Model		df	SS	Variance	Variance (%)	*p*
	**Microsatellites (11 loci)**						
Six regional groupings (5 DPS,	Among regions		5	501.42	0.2176	5.41	0.001
Saint John)	Among populations within regions		5	154.47	0.1716	4.27	<0.001
	Within populations		1,929	7,002.96	3.6304	90.32	<0.001
	Total		1,939	7,658.85	4.0196	100.0	
Seven regional groupings (5 DPS,	Among regions		6	532.78	0.2504	6.22	0.001
Saint John, Connecticut)	Among populations within regions		4	123.12	0.1423	3.54	<0.001
	Within populations		1,929	7,002.96	3.6304	90.24	<0.001
	Total		1,939	7,658.85	4.0231	100.0	
Seven regional groupings (4 DPS,	Among regions		6	528.71	0.1856	4.63	0.014
Saint John, Connecticut-Hudson,)	Among populations within regions		4	127.18	0.1930	4.81	<0.001
Delaware)	Within populations		1,929	7,002.96	3.6304	90.56	<0.001
	Total		1,939	7,658.85	4.0090	100.0	
Eight regional groupings (4 DPS,	Among regions		7	557.62	0.2453	5.52	0.009
Saint John, Connecticut, Hudson,)	Among populations within regions		3	98.28	0.1608	4.00	<0.001
Delaware)	Within populations		1,929	7,002.96	3.6145	90.46	<0.001
	Total		1,939	7,658.85	4.0206	100.0	
	**Mitochondrial DNA Control Region Sequence**						
Six regional groupings (5 DPS,	Among regions		5	281.28	0.1306	11.57	0.088
Canada)	Among populations within regions		6	147.63	0.2132	18.89	<0.001
	Within populations		1,466	1,150.21	0.7846	69.53	<0.001
	Total		1,477	1,579.12	1.1284	100.0	
Seven regional groupings (5 DPS,	Among regions		6	298.93	0.1540	13.61	0.011
Canada, Connecticut)	Among populations within regions		5	129.98	0.1931	17.06	<0.001
	Within populations		1,466	1,150.21	0.7846	69.33	<0.001
	Total		1,477	1,579.12	1.1145	100.0	
Seven regional groupings (4 DPS,	Among regions		6	288.60	0.0639	5.74	0.365
Canada, Connecticut-Hudson,)	Among populations within regions		5	140.32	0.2660	23.86	<0.001
Delaware)	Within populations		1,466	1,150.92	0.7846	70.40	<0.001
	Total		1,477	1,579.12	1.1145	1000.0	
Eight regional groupings (4 DPS,	Among regions		7	306.08	0.0895	8.01	0.094
Canada, Connecticut, Hudson,)	Among populations within regions		4	122.84	0.2435	21.79	<0.001
Delaware)	Within populations		1,466	1,150.21	0.7846	70.20	<0.001
	Total		1,477	1,579.12	1.1761	100.0	

Because of the genetic dissimilarity between the Connecticut River collection and others in the NYB DPS and its small effective population size, we treated it as a mixed stock and quantified the likely source(s) of its potential colonizers. Mixed stock analysis implemented in ONCOR indicated that approximately 50% of Connecticut River specimens originated in the Altamaha River within in the SA DPS (Table 10). Additional sources with moderate contributions included the James River in the CB DPS (23%) and Albemarle Sound in the Carolinas DPS (17%). It should be noted that the 95% confidence intervals around these estimates were broad.

**Table 10:** Population and DPS origin of juvenile Atlantic Sturgeon collected in the Connecticut River in 2014 based on Mixed Stock Analysis (MSA) estimates (95% CI) and Individual Based Assignment (IBA) testing implemented in ONCOR using microsatellite results at 11 loci and mtDNA control region sequence haplotypes.

Population (DPS)	MSA Estimate	95% CI	IBA Testing Assignment
Saint John (Canada)	0.021	(0.000, 0.064)	0.021
Kennebec (GOM)	0.000	(0.000, 0.000)	0.000
Hudson (NYB)	0.000	(0.000, 0.000)	0.000
Delaware (NYB)	0.000	(0.000, 0.000)	0.000
James (CB)	0.226	(0.000, 0.529)	0.222
Albemarle (CAR)	0.174	(0.000, 0.456)	0.200
Edisto (SA)	0.000	(0.000, 0.249)	0.000
Savannah (SA)	0.009	(0.000, 0.349)	0.000
Ogeechee (SA)	0.067	(0.000, 0.411)	0.040
Altamaha (SA)	0.502	(0.212, 0.773)	0.511

## Discussion

Although the Connecticut River probably once hosted a spawning population of Atlantic Sturgeon, there has been no evidence of successful natural reproduction there for many decades. Our collection of a moderate number (n = 64) of specimens in 2014 of which a subset (n = 45) were juveniles indicates that successful natural reproduction occurred in the Connecticut River in 2013. Furthermore, our genetic results on a subset of the 2014 collection (n = 45) indicate that the juveniles collected there were most likely offspring of a small number of recent colonizers from spawning rivers outside of the NYB DPS, mostly from the SA, CB, and Carolinas DPS. These results are contrary to expectations that recolonizers would most likely be migrants from proximal, not distant spawning populations. The low levels of microsatellite and mtDNA diversity exhibited by the Connecticut River collection is consistent with its much smaller *N*_*e*_ compared to juvenile collections from other population within the NYB DPS and coastwide.

We believe that the likelihood that the juveniles that we analyzed were spawned elsewhere and then migrated to the Connecticut River is negligible based on their size, our length frequency histogram (Fig 2), and their haplotypes/genotypes. Previous studies in other spawning rivers indicate that juvenile Atlantic Sturgeon do not emigrate into coastal waters until 70 cm [70] to 92 cm TL [7, 71] and 2–6 years of age [70]. All of the specimens analyzed genetically in our study were < 64 cm TL, even when collected in mid-fall, and based on our length frequency histogram (Fig 2) were 1 year of age. Dovel and Berggren [70] and Peterson et al. [72] showed that juveniles less than age-4 are commonly found in the Hudson River, but their abundance decreases after age-3 suggesting their mass emigration after age 3. Our conclusion that these juveniles were not migrants from elsewhere is also supported by our length frequency histogram (Fig 2) which exhibited a modal distribution from 35–55 cm TL; approximately the length where age-1 specimens were found previously in the Hudson [72], Altamaha [35] and Satilla rivers [2]. Furthermore, the most likely source of juvenile migrants to the Connecticut River would be the Hudson River, presumably the largest U.S. population and the most proximal. However, all the mtDNA haplotypes that we report in our Connecticut River collection (except one specimen with the A haplotype) were absent from the Hudson River (Table 3) and microsatellite analysis revealed highly significant allelic differences between our Hudson River and Connecticut River collections (Table 6). Furthermore, female-mediated gene flow between the Hudson River and Connecticut River was very low (0.537 migrants/generation) (Table 5).

Current thought holds that colonizers of newly available habitats or systems where populations were once extirpated are migrants from proximal extant sources. For example, Kinziger et al. [73] reported that an introduced population of speckled dace *Rhinichthys osculus* in northern California were derived from proximal and genetically similar populations rather than distant and genetically heterogeneous populations. Furthermore, it has been reported that successful colonizers usually exhibit robust levels of genetic diversity [74]. In contrast to these expectations, Connecticut River colonizers were from distant sources and exhibited depauperate levels of genetic diversity. It will be interesting to monitor the success of the 2013 Connecticut River juvenile cohort over future years.

Our analyses indicated that the 2014 Connecticut River juvenile collection was genetically distinct from all populations coastwide, including that in the proximal Hudson River in the NYB DPS and the Kennebec River in the adjoining GOM DPS. Identity of mtDNA haplotypes, rates of female-mediated gene flow, and phylogenetic analyses of the microsatellite data suggest that the Connecticut River juvenile collection was most closely related to populations within the SA DPS. We hypothesize that most of these newly discovered juveniles from the Connecticut River were descendants of a small number of colonizing parents probably of SA DPS ancestry, most likely the Altamaha River, whose population exhibits the highest frequency of the diagnostic E haplotype coastwide (39%) and which was found in 87% of Connecticut River juveniles (Table 3). But, it should also be noted that mixed stock analysis also indicated the presence of individuals of CB DPS, Carolinas DPS, and Canadian ancestry in the Connecticut River cohort.

Further evidence of the genetic uniqueness of the Connecticut River juvenile collection is its comparison to subadult collections from the Connecticut River made between 1989 and 2011 that were reported in [18]. Use of both the Exact G test and *F*_*ST*_ analyses indicate significant mtDNA haplotype and allelic frequency differences between the 2014 Connecticut River juvenile collection and the pooled sample of its subadults collected in earlier years (data not shown). It is likely that in 2013 the Connecticut River served as a spawning river for a genetically unique adult aggregation and hosted seasonal migrants of subadults from populations in all five DPS, but mostly from the Hudson River.

Because of the small number of mtDNA haplotypes in the Connecticut River collection and its reduced allelic diversity (Table 4) compared to other coastwide populations, we used COLONY to estimate family structure in the 2014 Connecticut River juvenile cohort (Table 7). These results were compared to those from three juvenile collections from the Hudson River and two from the Delaware River. We found the presence of 704 full-sibling dyads and only 11 families among the 45 specimens in the Connecticut River collection and only 18% of its specimens not associated with a full-sibling dyad. This compared to 51 families represented in the 2009 (n = 59) and 39 families represented in the 2011 (n = 49) Delaware River collection, respectively. Furthermore, there was an absence of full-sibling dyads in the 2011 (n = 30), 2013 (n = 35) and 2014 (n = 46) Hudson River collections, respectively. Thus, the relatedness of juveniles in the 2014 Connecticut River cohort was far greater and the number of families far less than in juvenile cohorts from the two other populations in the NYB DPS.

The extensive family structure in the Connecticut River cohort prompted us to compare its effective population size (*N*_*e*_) and effective number of breeders *N*_*b*_ with those of the juvenile collections from other populations in the NYB DPS. Not unexpectedly, *N*_*e*_ and *N*_*b*_ of the Connecticut River cohort were considerably smaller than those in the three juvenile cohorts from the Hudson River, the presumed largest population coastwide. Furthermore, *N*_*e*_ and *N*_*b*_ of the Connecticut River collection were even smaller than in two juvenile collections from the Delaware River, one of the smallest populations coastwide and at one time thought to be nearly extirpated. These results provided empirical evidence that the Connecticut River juveniles spawned there in 2013 were the likely offspring of a limited number of breeders.

How do the estimates of *N*_*e*_ that we determined in our study compare to those previously determined [75] based on ocean collections of subadult Atlantic Sturgeon empirically determined by mixed stock analysis and individual based assignment testing to be of Hudson River and Delaware River origin? Our estimates of 158.9 to 264.9 for the three Hudson River juvenile cohorts compares favorably with their estimate of 198 (95% CI; 171.7–230.7). However, our *N*_*e*_ estimates of 26.9 and 34.8 for the Delaware River juvenile cohorts are considerably smaller than their estimates of 108.7 (95% CI; 74.7–186.1) for Delaware River subadults (O’Leary et al. 2015). It is possible that their Delaware River estimates were inflated by the difficulty in accurately distinguishing between fish of Hudson River and Delaware River origins in the absence of mtDNA data.

Because of the genetic distinctiveness of the Connecticut River collection compared to the others from the NYB DPS and the likelihood that its juvenile aggregation was the offspring of a limited number of recent colonizers, we determined their likely population and DPS origin. Mixed stock analysis (MSA) and individual based assignment testing indicated that colonizers from the Altamaha River, Georgia, likely contributed the greatest percentage (50.2%) of offspring to the Connecticut River cohort, followed by the James River, Virginia (22.6%), and Albemarle Sound, North Carolina (17.4%) (Table 10). Thus, three of five DPS contributed to the Connecticut River juvenile aggregation but surprisingly, there was no evidence of contributions from either the proximal Hudson River nor the Delaware River in the NYB DPS. Although our previous genetic study [18] demonstrated that the Hudson River was the main contributor of subadults to Connecticut River and Long Island Sound collections, it also indicated the seasonal presence there of specimens from the SA, Carolinas, and CB DPS.

Along its extensive U.S. distribution, Atlantic Sturgeon is managed as five DPS based on their genetic discreteness, significance to the species as a whole, and differences in features such as habitat, climate and geology of spawning rivers as reported in Federal Register [10, 11]. Based on these criteria, the Hudson River and Delaware River populations were coupled into the NYB DPS which extends from Cape Cod, MA, to the Delaware-Maryland border. Although there has been considerable debate on the merits of this coupling based on differing trajectories in population abundances, varying threats, and the presence of a moderate frequency of unique mtDNA haplotypes in the Delaware River population [30]. Phylogenetic analysis of our microsatellite data showed strong evolutionary relatedness between the two populations. In contrast, our phylogenetic, STRUCTURE, and AMOVA analyses all indicate considerable genetic dissimilarity between the 2014 Connecticut River juvenile collection and the other two populations in the NYB DPS. If renewed natural reproduction continues to occur in the Connecticut River from broodstock that is genetically similar to that which produced the 2014 juvenile cohort or from colonizers from other distant DPS, the question arises as to which DPS the Connecticut River population should be assigned. Although geographically within the bounds of the NYB DPS, its closest genetic affinity is to populations within the SA, CB, and Carolinas DPS

This is at least the second example of an Atlantic Sturgeon population at one time thought to be extirpated exhibiting recent evidence of renewed successful natural reproduction. Unlike our random sampling in the Connecticut River, a directed and systematic sampling effort in the Satilla River, Georgia, detected juveniles (young-of-the-year and yearling) and thus evidence of natural reproduction in the third and final year of a three-year effort [2]. Similar to our results, the Satilla River juvenile collection was dominated by a single mtDNA haplotype and microsatellite analysis indicated that 39% of its individuals (n = 61) grouped into 14 full-sibling dyads. In our study, we found an even higher degree of genetic relatedness in our Connecticut River collection with 82% of individuals grouping into full-sibling dyads representative of 11 families. Unlike the current study, the predominant mtDNA haplotype in the Satilla River collection, haplotype D, was common in proximal populations within the SA DPS. Additionally, evolutionary analyses of the microsatellite data indicated that the Satilla River collection grouped with other populations in the SA DPS. This led Fritts et al. [2] to hypothesize that the Satilla River juvenile cohort were descendants of a cryptic aggregation of subadults of Satilla River ancestry that had resided in coastal waters for more than a decade and then, when mature, reinvaded the Satilla system and successfully began to reproduce there. In the current study, the overwhelming mtDNA haplotype in the Connecticut River, haplotype E, is common in populations in more southern DPS and was absent in proximal populations within the NYB DPS and GOM DPS. Furthermore, in the microsatellite UPGMA dendrogram, the Connecticut River collection was located outside of the clade containing populations in the NYB DPS. These findings suggest that recolonization of other rivers where Atlantic sturgeon historically occurred, but are now extirpated, may not always follow expectations as to their sources.

Our results should provide important new information and considerations for the effective federal management of Atlantic Sturgeon in two regards. First, NOAA recently proposed rules designating critical habitat for Atlantic Sturgeon in the GOM, NYB, and CB DPS in the Federal Register [76]. While critical habitats were proposed for the Hudson River and Delaware River within the NYB DPS, none were proposed for the Connecticut River. Our findings of successful reproduction there should provide justification for including critical habitats in the Connecticut River for protection under this designation. Second, because genetic population structure was one of the main criteria in designation of Atlantic Sturgeon DPS, our genetic characterization of the Connecticut River juvenile cohort pose the question as to whether it should be managed under the NYB DPS. While the results of our study should probably be viewed as an outlier, it still illustrates the unexpected potential pathways of gene flow in the context of Atlantic Sturgeon recovery. Furthermore, our results highlight the need for additional investigations of genetic structure in newly colonized rivers as well as in well-established populations.

## Conclusions

We report the first detection of successful natural reproduction of Atlantic Sturgeon in the Connecticut River in many decades. These were the smallest Atlantic Sturgeon collected in Connecticut waters in 28 years of sampling. Although our Connecticut River juvenile collection was genetically distinct from other collections coastwide, including the two other populations in the NYB DPS, it was surprisingly most closely genetically related to populations in geographically distant DPS. We recommend that directed sampling for juvenile Atlantic Sturgeon be conducted in the future in the Connecticut River to determine if successful spawning continues to occur, and if so, determine its genetic sources. It will also important to monitor the persistence of the 2013 cohort in the Connecticut River as they develop into subadults and to determine if additional successful spawning events reoccur.

## Supporting information

S1 Table Microsatellite allelic size data for each population. (DOCX)

S1 File Copyright release. (PDF)
