# Supplementary material for: Evidence of natural reproduction of Atlantic sturgeon in the Connecticut River from unlikely sources
Source: PLoS One. 2017 Apr 7;12(4):e0175085. doi: 10.1371/journal.pone.0175085 (PMC5384763; doi:10.1371/journal.pone.0175085)
Supplement: S1 Table — (DOCX) [file pone.0175085.s001.docx]

Connecticut River comparison 8-1-16

AoxD44, LS54, AoxD188, AOX45D3, LS39, LS68, AoxD165, LS19, Aox23, AoxD170, Aox241

pop

AO0160 , 131159 186186 310310 000000 129129 147151 206214 150150 097097 144144 210222

AO0176 , 143155 166186 310310 130130 129129 155163 202210 134150 097118 152164 222270

AO0177 , 127155 186186 310342 130145 129129 147151 202202 134150 097118 136148 202210

AO0178 , 155155 186186 310310 130145 129135 147151 202210 134150 097097 148148 198210

AO0179 , 155175 186186 310342 130130 129129 151159 202202 134134 097097 136144 206210

AO0180 , 127155 186186 310346 133145 129129 147155 202202 134150 097118 144148 194206

AO0181 , 155155 186186 342358 130145 129129 147147 198206 134150 118118 140164 210222

AO0182 , 155159 186186 310322 145145 129129 151159 210226 150150 097118 144152 202214

AO0184 , 155155 186186 310342 112130 129129 147151 202202 134134 100118 140148 194242

AO0186 , 143155 186186 310330 130145 129129 151159 202202 150150 097118 144144 194234

AO0187 , 155155 186186 310310 133145 129129 151155 202202 134134 097118 140144 186194

AO0188 , 143175 186186 310342 130130 129129 151155 202206 134134 097097 136136 206206

AO0189 , 143187 186186 310310 130130 129129 151155 210210 134150 118118 148148 206218

AO0190 , 155187 166186 310310 112112 129129 135151 198202 134150 097097 144148 194222

AO0191 , 187195 166186 310310 130130 129129 151159 198202 150150 100118 136152 198214

AO0192 , 143155 186186 346358 112139 129129 147155 198226 150150 118118 140144 202210

AO0193 , 143155 166186 143155 130142 129129 155159 202202 150150 118121 144152 202206

AO0194 , 163187 186186 310358 130145 129135 155159 202218 134170 097097 140148 202222

AO0195 , 191195 186186 326346 130151 129129 147151 202202 134150 118118 144164 198202

AO0196 , 143155 186186 310314 142145 129129 151159 202202 134134 097097 140144 210242

AO0197 , 143187 186186 310314 130139 129129 135147 202226 150170 100118 148156 202206

AO0198 , 143175 186186 310346 145145 129129 135147 198202 134170 097118 156156 218222

AO0199 , 175175 166186 310342 115130 129129 147155 206218 150150 100118 136144 202206

AO0200 , 155159 186186 310310 130145 129129 151151 202210 134134 097118 136148 218222

AO0201 , 159187 186186 310310 130145 129129 147159 202202 134150 097097 144152 198222

AO0202 , 143175 166186 310346 130148 129129 151155 202206 134134 097100 156164 202214

AO0203 , 143195 186186 310342 130145 129129 151159 198218 134150 100118 144152 206218

AO0204 , 155155 166186 310310 130145 129129 151159 202218 150150 097118 148152 202206

AO0205 , 143155 186186 310310 130130 129129 147155 198206 134150 136136 140144 226246

AO0206 , 155155 186186 298310 130130 129129 147155 218218 134150 097097 144152 202206

AO0207 , 143187 186186 310346 130145 129129 151151 202206 134134 097097 144148 214222

AO0387 , 155163 186186 310330 145145 129135 147155 202218 150150 097097 144144 202206

AO0388 , 143143 186186 310310 130136 129129 151155 202202 134150 097097 136152 194202

AO0389 , 143155 166186 310310 115130 129129 147155 206218 134150 100118 148148 218222

AO0390 , 127155 186186 310346 139145 114129 147155 202202 134150 121136 144152 186194

AO0391 , 159175 166186 310310 112112 129129 135155 202202 134150 097100 136148 194222

AO0392 , 155163 166186 342358 130145 129135 147155 202202 150170 097118 136140 202218

AO0393 , 143155 186186 314314 139145 129129 135147 202202 134150 097097 148148 202210

AO0394 , 155187 186186 310346 130130 129129 151155 202202 134150 097100 144156 222222

AO0395 , 155195 186186 314358 121130 129129 147151 202202 134150 118118 148156 198202

AO0396 , 155187 186186 310310 130145 129129 147147 202206 134134 097097 136148 202222

AO0397 , 155159 186186 310346 130139 129129 143151 202202 134170 097118 148148 186202

AO0398 , 155155 186186 310310 130145 129129 159159 202202 134134 097118 136156 198202

AO0399 , 127143 186186 310346 130133 129129 151155 202202 150170 097136 140144 194206

AO0400 , 143159 166186 310346 115115 129129 151159 202206 150150 097118 144144 194202

AO0401 , 155195 166186 310358 112130 129129 151155 202202 150150 097097 136164 194198

AO0402 , 155175 186186 310310 130145 129129 147151 202210 134134 097118 148152 206210

AO0403 , 175195 166186 310358 112130 129129 151151 202206 134134 118136 136152 194198

AO0404 , 155187 186186 310346 130130 129129 151155 202206 150150 118118 144144 198230

AO0405 , 143191 186186 310346 112139 129129 143147 202226 150150 118118 140148 202246

AO0406 , 143175 166186 310358 130130 129129 135159 202218 150150 097118 140152 194202

AO0407 , 143175 166186 310342 133151 129129 147155 202218 134150 097118 136148 206218

AO0408 , 155155 186186 310310 112145 129129 147147 202218 150150 097097 136148 202206

AO0409 , 127155 166186 310346 130142 129129 155159 202202 134134 118121 144156 202206

AO0410 , 143155 186186 310342 130130 129129 147151 198198 134134 097097 140148 218222

AO0411 , 143187 166186 310346 115130 129129 151155 202206 134150 097118 144144 198202

AO0412 , 143159 186186 310310 121145 129129 147167 202210 134150 103118 136144 206250

AO0413 , 155159 186186 310310 130145 129129 151155 198206 134134 097118 136156 222230

AO0414 , 143143 186186 310310 130145 129129 151151 206218 134170 097118 136140 202206

AO0415 , 155175 186186 310310 112112 129129 159159 202206 134150 118136 144144 218254

AO0416 , 143155 166186 310310 130130 129129 151151 198206 134150 100118 148152 194218

AO0417 , 143155 186186 310310 115130 129129 151155 218218 134150 100118 144148 206218

AO0419 , 155175 186186 314346 139145 129129 147155 202226 134150 118118 144148 194202

AO0420 , 143155 166186 310314 130130 129129 147155 210218 134150 097097 136144 206218

AO0421 , 143159 186186 310310 121130 129129 147167 194194 134150 100100 000000 000000

AO0422 , 143155 166186 310346 130130 129129 155155 202202 134134 097136 144144 194222

AO4808 , 143143 186186 310310 112139 129129 147159 198202 150150 097118 136156 202218

AO4809 , 143187 186186 310310 130145 129129 147151 202210 134134 100118 148156 218218

AO4810 , 143175 166186 310358 142145 129129 155155 198218 000000 097097 144144 202222

AO4811 , 175191 166186 342346 112112 129129 147159 202202 134134 097097 148152 194214

AO4812 , 155163 166186 310346 145145 129129 147159 210210 134134 097100 144148 206210

AO4813 , 155159 186186 310346 142142 129129 155155 202202 150150 127136 136144 194250

AO4814 , 155155 186186 310342 130139 129129 147159 218226 134150 097118 136148 202206

AO4815 , 187191 166186 310310 112130 114129 135159 202226 134150 097118 144144 198202

AO4816 , 127155 186186 310310 133145 129129 147155 202202 150150 097136 140144 186234

AO4817 , 127143 186186 310310 130130 129129 155159 194206 150170 118118 136148 202258

AO4818 , 155159 166186 310310 130130 129129 151159 202202 150150 097100 148152 194258

AO4819 , 175187 186186 310310 115133 129129 151159 198206 134134 097097 148152 202214

AO4820 , 175191 186186 310314 112130 129129 147155 206218 150150 097097 148148 206222

AO4821 , 155155 186186 342358 130139 129129 147147 206210 134150 097118 136164 210222

AO4822 , 155191 166186 310310 139145 129135 147159 210218 134134 097118 136164 202210

AO4823 , 155155 186186 310358 121145 129129 147159 210226 134150 097097 136164 202210

AO4824 , 143155 186186 322346 130130 129129 155155 198218 134134 097118 136152 206222

AO4825 , 127155 186186 310310 130145 129129 143151 202218 134150 103121 136148 194202

AO4826 , 143187 186186 310346 130130 129129 147159 202202 134134 097118 148152 198202

AO4827 , 155159 186186 310346 130139 129129 147151 198202 134134 097097 148152 222222

AO4828 , 127155 186186 310346 142145 129129 147155 202202 150150 097118 144164 194214

AO4829 , 155175 186186 310310 130145 129129 151155 206218 134150 118136 136148 202210

AO4830 , 155155 186186 310310 133145 129129 147151 198202 134170 097121 136140 202234

AO4831 , 143179 186186 310346 130145 129129 151151 202210 134134 097118 148148 194194

AO4832 , 155155 186186 310342 145145 129129 159159 202206 150170 097097 140148 194222

AO4833 , 155159 186186 310310 130130 129129 159163 202218 134150 097097 136152 202222

AO4834 , 143155 186186 346346 130130 129129 147151 202202 134150 094100 148156 206234

AO4835 , 155187 186186 310310 133142 129129 147147 202202 150150 097118 144148 222234

AO4836 , 143155 186186 310310 130130 129129 155155 202202 150170 118118 148156 194198

AO4837 , 127155 166186 322346 121145 129129 155167 202202 150150 097136 144156 222250

AO4838 , 155159 186186 330342 130130 129129 151159 202218 134150 097100 136152 206218

AO4839 , 143155 166186 310310 133145 129129 151159 202210 150170 097097 152152 194206

AO4840 , 155159 166166 310310 139142 129129 151159 198202 134150 097097 136156 222262

AO4841 , 143191 186186 310310 139139 129129 147159 202226 150170 100118 148156 202206

AO4842 , 143155 166186 310314 112130 129129 155159 194218 150150 097097 144148 202218

AO4843 , 143155 166186 310310 130130 129129 151155 202218 134134 097100 136148 206222

AO4844 , 127155 186186 310310 145145 129129 155155 202202 150150 097121 140144 186234

AO4845 , 155187 166186 310342 130130 129129 147151 202202 150150 097097 136136 246258

AO4846 , 155175 186186 298342 130130 129129 151159 206206 150150 097097 148152 194218

AO4847 , 155191 186186 310342 130130 129129 147159 202202 134150 097100 144148 194206

AO4848 , 155159 186186 310310 130130 129129 151159 198202 134150 094097 144148 202222

AO4849 , 155191 186186 310342 130130 129129 147147 202210 150150 097097 136156 210246

AO4850 , 155187 186186 310342 130130 129129 151159 202210 150150 100121 136144 206206

AO4851 , 143187 186186 342346 130145 129129 151151 202202 150150 097097 152156 218234

AO4852 , 155159 186186 322346 130130 129129 151155 194202 134150 097097 148148 194222

AO4853 , 155155 186186 310310 139145 129129 159159 218218 134134 100118 136164 202206

AO4854 , 143187 166186 310310 130130 129129 151151 202218 150150 097100 136148 194206

AO4855 , 155155 186186 310310 130133 129129 155159 202210 134150 118118 152156 198206

AO4856 , 155159 186186 310310 130130 129129 135151 202202 134150 118118 136148 206254

AO4857 , 143187 166186 310346 115115 129129 155159 198218 134150 097118 144164 202222

AO4858 , 143155 186186 310346 130130 129129 155159 202206 150150 097100 144144 210222

AO4859 , 127155 186186 298342 130130 129129 151155 202206 150150 097100 148152 194202

AO4860 , 155175 166186 310310 115145 129129 155159 198218 134150 100118 144164 202222

AO4861 , 159187 166186 310346 142145 129129 155167 202202 150150 127136 136144 194262

AO4862 , 155155 186186 342346 130145 129129 151159 194202 134150 097097 144156 222254

AO4864 , 143159 166186 310358 130142 129129 155159 202202 134150 097100 148164 198258

AO4865 , 191195 186186 314346 139139 129129 147155 202226 134150 097097 144148 206206

AO4866 , 143155 186186 310310 130130 129129 135135 202202 150150 094136 140156 194202

AO4867 , 143143 186186 274310 130145 129129 147159 202210 150170 097097 144148 214226

pop

AO3830 , 127171 186186 310310 000000 000000 000000 198202 134150 097106 000000 000000

AO3831 , 163187 186186 310342 139145 129129 135155 206210 150150 097097 136152 182210

AO3832 , 159163 166186 274310 112139 129129 151159 198206 127150 097097 144144 210234

AO3833 , 167167 186186 310310 145151 129129 135155 202206 134150 097121 144156 238254

AO3834 , 171179 186186 310334 127151 129129 143151 206218 134134 097118 144156 210210

AO3835 , 143167 186186 310310 124124 129129 135155 198210 134134 100118 140156 210270

AO3836 , 155163 166166 310342 130145 129129 147155 202214 150150 100121 140144 206238

AO3837 , 143187 186186 310342 130130 129129 151155 198202 134150 097118 144144 190270

AO3838 , 127143 186186 310310 145148 129129 151155 202206 150150 097109 148164 206234

AO3839 , 143187 166186 310310 142142 129129 151151 202214 127150 097106 144156 202218

AO3840 , 163191 186186 342342 139157 129129 135147 206206 150150 097100 148152 210210

AO3841 , 127187 166186 310310 130151 129129 143155 218226 150150 097106 156160 210254

AO3842 , 143187 186186 310342 139142 129129 151155 198202 134150 100118 152156 210270

AO3843 , 167171 186186 310314 130142 129129 155155 198206 134150 118118 156156 210270

AO3844 , 143163 186186 310310 133145 129129 135147 202202 134134 097127 144156 190206

AO3845 , 167171 186186 274310 133145 129129 147151 202226 150150 097100 156160 210254

AO3846 , 163175 166186 310338 142151 129129 143159 202218 134134 097100 144164 202246

AO3847 , 163175 166186 274310 118145 000000 151159 202202 134150 097097 140144 234254

AO3848 , 167167 166186 274314 139151 129129 143159 202206 150150 097118 144144 210238

AO3849 , 163187 186186 310310 142145 129129 135151 202210 150150 100136 156156 182198

AO3850 , 163191 186186 306310 130145 129129 135147 202206 134150 097109 152156 206226

AO3851 , 171171 186186 310342 169169 129129 135155 198202 134150 118118 136144 206274

AO3852 , 127163 186186 310310 145169 129129 135147 202206 134150 097097 144156 206226

AO3853 , 127163 166186 310310 136151 129129 155155 210226 134150 097100 144160 254254

AO3854 , 163163 166186 274310 130139 129129 155159 206210 134150 097100 140144 210210

AO3855 , 143171 186190 310310 148157 129129 135155 206218 134150 097118 140164 206218

AO3856 , 143143 186186 310310 145145 129129 155155 202206 134150 097127 144156 202214

AO3857 , 127163 166190 274334 127139 129129 151159 202206 134150 097097 144144 230238

AO3858 , 127171 186186 274310 148157 129129 151159 206218 134150 109118 144144 218254

AO3859 , 127163 186186 310310 133139 129129 143147 206226 134150 097127 144156 210250

AO3860 , 143167 166186 310310 133139 129129 143147 206226 150150 097127 144156 202250

AO3861 , 127127 186190 310310 145157 129129 151159 198206 134150 097109 140164 234254

AO3862 , 127175 166186 298314 139169 129129 151155 202226 150150 097136 156156 202222

AO3863 , 163163 186186 310310 130145 129129 151159 202226 150150 109118 140144 190266

AO3864 , 159163 166186 310310 112130 129129 155159 198206 127150 097097 144144 198210

AO3865 , 163171 186186 310342 145145 129129 147155 202202 134150 100118 152156 206210

AO3866 , 143167 186186 310310 130139 129129 155159 202206 134134 097118 136144 194210

AO3867 , 167167 166186 310314 124145 129129 151155 194210 134134 097118 140156 210266

AO3868 , 143171 186186 310310 145145 129129 151151 202218 134150 097097 144164 194218

AO3869 , 127127 166186 310310 130148 129129 151155 202218 134134 109118 144164 210218

AO3870 , 163175 186186 310310 145145 129129 135155 202206 134150 097118 156156 210226

AO3871 , 143167 166186 310310 130139 129129 155159 202218 134150 097118 144144 194246

AO3872 , 159171 186186 310310 112139 129129 147151 198206 150150 097097 144148 198210

Pop

AO5281 , 127171 186186 314326 118133 129135 155163 194218 153153 100103 148160 194242

AO5282 , 127171 186186 314322 118133 129129 155163 194218 153153 097100 156160 194242

AO5283 , 127127 186186 314322 127133 129135 147163 202214 147150 097100 148160 186198

AO5284 , 127171 186186 314326 118133 129135 151163 194218 150153 097100 148160 194242

AO5285 , 127171 186186 314326 118142 129129 155163 190202 153153 100103 156160 198230

AO5286 , 127171 186186 314326 118133 129135 151163 190194 150153 103118 156160 194230

AO5287 , 127171 186186 314326 118133 129135 151163 190194 153153 103118 156160 194230

AO5288 , 127171 186186 314322 118142 129129 151163 190194 153153 103118 156160 194230

AO5289 , 127175 186186 310310 130145 129129 147155 202202 150150 097100 136144 202222

AO5290 , 131171 186186 314314 121145 129129 151163 190202 150153 100103 156160 198230

AO5291 , 127171 186186 314322 118133 129135 155163 190202 150153 097118 148160 198230

AO5292 , 127171 186186 314322 118133 129129 151163 202218 150153 100103 156160 198242

AO5293 , 127171 186186 314326 118133 129129 155163 202218 150153 100103 156160 198242

AO5294 , 127171 186186 314326 118133 129129 155163 190194 153153 097100 156160 194230

AO5295 , 127171 186186 314322 118133 129135 151163 202218 153153 097100 148160 198242

AO5296 , 127171 186186 314326 118133 129129 000000 202218 150153 100103 148160 198242

AO5297 , 127171 186186 314326 118133 129129 155163 194218 150153 097100 156160 194242

AO5298 , 127171 186186 314326 118142 129135 151163 202218 150153 103118 148160 198242

AO5299 , 127171 186186 314322 118142 129135 151163 194218 150153 097118 148160 194242

AO5300 , 127171 186186 314326 118133 129129 151163 190194 150153 097118 156160 194230

AO5301 , 127143 186186 314322 127142 135135 147163 202210 150153 097100 156160 198250

AO5302 , 127171 186186 314322 118142 129135 151163 194218 153153 097100 148160 194242

AO5303 , 127171 186186 314322 118142 129135 151163 202218 150153 097100 148160 198242

AO5304 , 127171 186186 314326 118133 129129 155163 190202 153153 097100 148160 198230

AO5305 , 127171 186186 314326 118142 129129 151163 194218 153153 097100 156160 194242

AO5306 , 127127 186186 314348 127133 135135 147163 194214 150150 097100 148152 186194

AO5307 , 127171 186186 314326 118142 129129 155163 202218 153153 097100 148160 198242

AO5308 , 127171 186186 314326 118133 129135 155163 190194 153153 103118 156160 194230

AO5309 , 127171 186186 314322 118142 129135 151163 190194 150153 103118 148160 194230

AO5310 , 127171 186186 314326 118133 129129 155163 194218 150153 100103 156160 194242

AO5311 , 127171 186186 314322 118142 129129 155163 202218 150153 100103 148160 198242

AO5312 , 127171 186186 314326 118142 129135 155163 190202 153153 103118 148160 198230

AO5313 , 127171 186186 314326 118142 129135 155163 190194 153153 100103 148160 194230

AO5314 , 127171 186186 314322 118133 129135 155163 190202 150153 100103 156160 198230

AO5315 , 127171 186186 314326 118133 129135 155163 190194 153153 100103 148160 194230

AO5316 , 127127 186186 314322 127142 135135 155163 194214 134153 097118 156160 186194

AO5317 , 127171 186186 314322 118142 129135 155163 190194 153153 097118 156160 194230

AO5318 , 127171 186186 314322 118133 129135 155163 202218 153153 097118 148160 198242

AO5319 , 127143 186186 314346 127133 135135 155163 202214 134150 097100 148152 186198

AO5320 , 127171 186186 314322 118133 129129 155163 202218 150153 097100 156160 198242

AO5321 , 127171 186186 314322 118142 129129 151163 202218 153153 103118 156160 198230

AO5322 , 127171 186186 314326 118142 129135 155163 190194 153153 103118 148160 194230

AO5323 , 127171 186186 314322 118142 129129 151163 190194 150153 097118 156160 194230

AO5324 , 127171 186186 314326 118142 129135 155163 190202 150153 103118 156160 198230

AO5325 , 127171 186186 314322 118133 129129 151163 190202 153153 097118 156160 198230

pop

AO4404 , 167183 190190 274346 136139 129129 143147 206206 150150 097115 144152 210234

AO4405 , 127175 186186 274314 130148 129129 147151 206210 134150 097100 136164 198206

AO4406 , 127127 186186 310338 139145 129129 143155 198206 127153 097100 140144 206222

A04407 , 163187 186186 310322 136139 129129 143147 206210 153170 097106 144164 206222

AO4408 , 175183 186186 310310 130139 129129 147151 198202 134170 097097 144148 214230

AO4409 , 127167 166190 274310 145145 129129 143151 206206 150150 097100 148164 210222

AO4410 , 127179 186186 334346 121136 129129 147159 202206 150153 097118 144156 206234

AO4411 , 127127 166190 310322 130148 129129 151159 206218 127134 097100 148168 210210

AO4412 , 127127 186186 274310 139148 129129 147147 194202 134150 097097 140148 190258

AO4413 , 143163 186186 294310 139142 129129 147147 194206 134153 100100 140164 186266

AO4414 , 127159 186186 274310 130142 129129 147151 198206 134150 097097 144152 198242

AO4415 , 127155 186186 310314 136142 129129 151155 194218 134150 100100 144164 210266

AO4416 , 159163 186190 310310 130148 129129 147151 202214 150168 097118 136164 214234

AO4417 , 127163 186186 294310 130145 129129 147147 198202 150150 097100 136144 222238

AO4418 , 143187 186186 310310 127148 129129 147155 194206 150150 097136 144148 210266

AO4419 , 163167 186186 310310 139145 129129 147151 202206 134134 100115 148164 206214

AO4420 , 155159 186186 310318 139145 129129 143147 198218 134150 097136 140144 202214

AO4421 , 163171 186186 310310 000000 129129 143143 218226 127134 106106 140164 000000

AO4422 , 143159 186186 298310 115145 129129 155155 198206 127150 097097 136144 198242

AO4423 , 127127 186190 310322 133142 129129 155155 202206 150150 103115 144148 210234

AO4424 , 175183 186186 294310 133142 129129 147147 194202 134150 097097 144164 230270

AO4425 , 127159 186190 274322 124130 129129 147151 206218 134134 097100 152164 206218

AO4426 , 127127 166186 334346 139142 129135 143151 206218 170170 097127 156168 206210

AO4427 , 127187 186186 266322 133139 129129 147155 206210 150170 100100 136148 210218

AO4428 , 155163 166166 310310 139142 129129 143151 198202 150170 097100 144164 214222

AO4429 , 155187 186186 310334 136139 129129 143147 194202 134153 106118 144144 190266

AO4430 , 127179 166186 310314 139148 129129 147151 198206 134170 097127 144144 206262

AO4431 , 127187 166186 310310 124142 129129 143147 202206 134153 100100 144152 202234

AO4432 , 127143 186186 274310 127148 129129 147151 206218 150150 097100 148152 214214

AO4433 , 127127 186186 310322 133148 129129 155167 000000 150150 100100 148148 210226

AO4434 , 163187 186186 266314 133142 129129 147147 194206 134150 097100 140148 210270

AO4435 , 155163 186186 310322 124139 129129 147159 206218 134150 100106 136148 206210

AO4436 , 159187 166186 310346 127139 129129 143151 194206 134134 097127 144168 206270

AO4437 , 175179 166186 298310 115139 129129 147155 000000 150150 097100 136144 210222

AO4438 , 139163 166186 310310 136142 129129 147147 206206 134134 097097 144156 198214

AO4510 , 127175 166186 290322 139142 129129 147147 198206 127134 097100 136144 222226

AO4511 , 155187 186186 310358 145148 129129 151151 202210 127134 097097 140144 210230

AO4512 , 127155 166186 294310 139142 129129 147155 206218 134150 097118 140164 210214

AO4513 , 127155 186186 274358 133148 129129 143151 206218 134150 097115 156156 210254

AO4514 , 127167 186186 310310 000000 129129 131143 198198 127127 097136 144168 222230

AO4515 , 127175 186186 310310 139142 129129 143143 202206 134150 097097 144148 202210

AO4516 , 127127 186186 294358 145145 129129 147147 218218 150153 097100 140144 198206

AO4517 , 127163 186190 310310 130145 129129 147155 202202 127150 100100 144144 234250

AO4518 , 159175 186186 310358 133142 129129 147151 206206 150150 097115 144156 214222

AO4519 , 143175 186186 310314 139145 129129 147151 206206 127134 097127 144144 198218

AO4520 , 155167 186186 310310 121142 129129 143151 206214 134150 100100 144148 214218

AO4521 , 127187 166186 294310 139148 129129 151155 206206 134150 097115 140144 206254

AO4522 , 127187 186186 310310 124139 129129 151151 194222 134134 097115 136148 206266

AO4523 , 155163 000000 294310 139145 129129 151151 202206 134134 106127 140144 198222

AO4524 , 127155 186186 310318 121142 129129 151159 198202 134134 097100 140144 198210

AO4525 , 127127 186186 294314 142148 129129 147151 198206 134134 097100 144144 222230

AO4526 , 143175 166186 310358 139145 129129 155167 206222 134134 097118 144156 198214

AO4527 , 127159 186186 278310 139148 129129 143147 206206 127150 097127 144144 198214

AO4528 , 127155 166186 294310 139142 129129 143155 202202 134150 106115 140144 218222

AO4529 , 127183 186186 298334 139142 129129 147155 206218 134150 097097 136156 210242

AO4530 , 155163 166186 310310 136148 129129 143143 198206 127150 097097 140148 214274

AO4531 , 127187 166186 294310 130136 129129 143147 206206 134150 100106 140148 222226

AO4532 , 127127 186186 310310 139142 129129 143147 206210 134150 097097 144144 198210

AO4533 , 127143 186186 310310 124139 129129 147151 206206 127134 115127 136144 198198

AO4534 , 143143 186186 294310 130142 129129 151151 202206 134153 097115 140148 214226

AO4535 , 127127 166186 294298 142145 129129 147147 202206 134170 097097 136144 186198

AO4536 , 127187 166186 294310 142145 129129 143147 194206 134150 100127 144164 186266

AO4537 , 187187 186186 310310 127148 129129 143151 194214 134134 097097 144144 226266

AO4538 , 127127 186190 310322 139145 129129 143155 194198 134134 097100 136144 198274

AO4539 , 163163 186186 310310 130139 129129 147151 194210 134134 097097 160164 230258

AO4592 , 159163 166186 310346 142148 129129 147151 206218 150153 097118 140148 210210

AO4593 , 163187 186186 310322 139145 129129 147155 206206 150170 097118 136144 206210

AO4594 , 127127 166186 310314 139145 129129 151151 194198 134150 100127 140164 266270

AO4595 , 139159 186186 310310 139139 129129 151155 214218 134150 097100 136144 218226

AO4596 , 179187 166186 318350 145148 129129 147147 198206 134134 097103 140140 202210

AO4597 , 143175 186186 298298 115130 129129 143167 202202 134134 097100 140148 198222

AO4598 , 159187 166186 310314 115142 129129 143151 194194 150150 097097 144156 266266

AO4599 , 159187 186186 310310 127145 129129 155155 202218 134150 097127 140148 198202

AO4600 , 127143 186186 274310 127139 129129 147147 206206 127150 100100 148148 198214

AO4601 , 155187 186186 310342 127139 129129 147153 198210 150150 097115 156164 198222

AO4602 , 127159 186186 310334 136142 129129 147155 218218 134150 097118 140156 210218

AO4603 , 127187 186186 266310 130148 129129 147147 206222 127150 097097 148168 206226

AO4604 , 127127 186186 314326 133142 129129 147159 194218 134150 097100 144148 210222

AO4605 , 127155 186186 310346 142145 129129 143151 206218 134170 097115 148164 198214

AO4606 , 163187 186186 310310 136142 129129 147155 206206 134150 097097 136144 210214

AO4607 , 143187 186190 310334 139142 129129 139147 202206 127150 100100 144148 198210

AO4608 , 143143 166166 294322 121148 129129 147147 206214 127134 097106 144148 206250

AO4609 , 143163 186186 274310 130133 129129 147155 198206 127150 097100 144148 214270

AO4610 , 127159 186186 274310 130142 129129 151155 206218 134134 097097 148152 206214

AO4611 , 143183 186186 294310 130145 129129 147155 206218 134134 106127 140160 210214

AO4612 , 155187 186190 274322 136139 129129 147147 198218 127127 097118 144164 198242

AO4613 , 127127 186186 310310 136139 129129 139147 194206 134150 100103 140144 214270

AO4614 , 159179 166186 310310 136139 129129 143147 202206 127150 097097 136144 198222

AO4615 , 155175 186186 294346 115142 129129 155167 194206 134134 097100 164164 210266

AO4616 , 127175 186186 274310 136142 129129 147147 198206 134134 097100 144148 210214

AO4617 , 127163 186186 294310 139142 129129 143147 194206 153168 097097 136144 214266

AO4618 , 143159 166186 274310 136142 129129 147151 202206 134168 097100 144156 202202

AO4619 , 179187 186186 274294 130145 129129 155155 198218 134150 097100 144152 198234

AO4620 , 143175 166186 294310 130142 129129 143147 202206 150150 100115 144164 202230

AO4621 , 127175 186186 274310 127142 129129 147151 202206 127134 097118 140164 222262

AO4622 , 127127 186186 310314 142148 129129 143155 202218 134150 100115 164164 202202

AO4623 , 127155 186186 294310 142145 129129 151151 206218 134150 097100 140144 198210

AO4624 , 127187 186186 274310 127130 129129 147151 214214 134150 097100 148164 214226

AO4625 , 159179 186186 310310 142145 129129 143143 198202 134134 097106 144144 202222

AO4626 , 155163 186186 310322 136136 129129 147147 202218 127134 097100 148164 206210

AO4627 , 127155 186186 310310 127142 129129 147151 194202 134150 097106 144144 202266

AO4628 , 127187 186186 294310 133142 129129 151155 194198 127134 103127 144164 234266

AO4629 , 159163 186186 310310 133136 129129 147151 198206 127134 097097 144148 214226

AO4630 , 127127 186186 310310 145148 129129 143147 194202 134150 106127 136144 222222

AO4631 , 127155 186190 294310 139142 129129 143143 206214 134150 118136 140144 214214

AO4632 , 159187 166186 274310 142142 129129 147151 198206 134150 097097 136164 210242

AO4633 , 143187 186186 274274 142142 129129 143151 206210 134150 097097 152152 206222

AO4634 , 155183 190190 310310 142142 129129 147151 202206 134134 097118 148160 202234

AO4635 , 127187 186186 294310 136139 129129 155155 198218 150170 100106 140144 206210

AO4636 , 127179 166186 310346 139145 129129 143151 202206 127150 100106 144168 210250

AO4637 , 127175 186186 310314 130142 129129 147151 206214 134134 097118 144148 206218

pop

AO3158 , 143155 166166 310310 139148 129129 143147 206210 134150 097127 140148 214230

AO3159 , 127171 186186 310318 139148 129129 147147 206214 150150 097097 144144 206226

AO3160 , 143143 186186 294326 139139 129129 147155 194218 134170 097100 140144 218270

AO3161 , 127167 186186 310326 136139 129129 147151 206206 134134 097106 144144 190206

AO3162 , 127127 166186 310358 136139 129129 147147 194210 134150 097106 144148 222266

AO3163 , 143159 186186 310326 139142 129129 147147 194202 134134 097118 136144 206266

AO3164 , 143159 186186 310326 139142 129129 147155 202206 150150 097127 144164 206210

AO3165 , 159167 166186 310314 130136 129129 147155 202206 150150 097118 140164 210234

AO3166 , 159183 186186 274310 127139 129129 131147 202206 127150 100118 164164 210226

AO3167 , 127143 166186 310326 136142 129129 147155 206206 150170 106118 144164 214214

AO3168 , 127143 166186 310358 136142 129129 147155 202206 134134 106118 144144 206214

AO3169 , 127127 166186 310358 142145 129129 147155 202210 134150 100118 144144 206222

AO3170 , 127143 186190 310326 142142 129129 147155 202206 134134 097097 144144 190206

AO3171 , 143143 166186 274346 130142 129129 143147 210214 134134 118118 140148 234250

AO3172 , 127159 186186 310346 136142 129129 147151 194202 134150 097097 144168 222266

AO3173 , 143159 166186 310310 130139 129129 139147 202206 134134 097097 144144 190230

AO3174 , 159171 186186 310310 127139 129129 147151 206206 134150 097106 144160 206210

AO3175 , 127163 166186 310334 136142 129129 151151 202210 150150 097106 156164 222250

AO3176 , 127159 166186 326358 136142 129129 147147 206206 134150 097106 144144 190214

AO3177 , 127171 166166 318358 136142 129129 147147 202206 134150 097106 144144 214214

AO3178 , 127127 166186 310310 136139 129129 143147 194210 150150 097100 148164 222266

AO3179 , 127159 186186 310346 139148 129129 147147 202202 134150 100106 148156 190234

AO3180 , 127143 166186 294346 139145 129129 147151 198222 134150 097100 140156 210218

AO3181 , 159175 186186 326342 139142 129129 147151 206206 134134 100127 144156 190218

AO3182 , 159171 166186 310326 139142 129129 143151 194194 134150 100127 144148 266266

AO3183 , 183183 166166 274310 127136 129129 151163 198202 134134 097097 148164 218234

AO3184 , 143143 166186 310326 139142 129129 143147 206206 134150 100118 144144 214250

AO3185 , 127127 166166 310314 136136 129129 151159 194206 134134 106127 140164 214266

AO3186 , 159163 166186 274310 136148 129129 143147 202206 134134 097097 148164 210210

AO3187 , 159175 186186 310326 139139 129129 139147 194206 134134 097127 144148 218218

AO3188 , 127159 166186 314322 139142 129129 147151 194206 150170 097100 156164 214266

AO3189 , 159167 186186 274310 121139 129129 131147 202206 127134 097097 164164 210214

AO3190 , 159171 186190 310326 139139 129129 147147 194194 134150 097100 144148 266266

AO3249 , 143143 186190 326326 142142 129129 147151 206206 134134 097097 144144 190190

AO3250 , 127159 186190 322326 139142 129129 139151 206206 134134 097097 136144 190234

AO3251 , 159167 186186 274310 121136 129129 131147 202206 127150 097118 164164 210226

AO3252 , 155159 166186 294326 139139 129129 147155 206214 134170 097103 140144 190214

AO3253 , 143143 186190 294310 142142 129129 143151 206214 134150 097103 140144 190214

AO3254 , 127159 166186 310322 127136 129129 147151 206210 134134 097100 160164 210222

AO3255 , 143159 186186 310326 139142 129129 151151 202206 134134 100118 144164 190222

AO3256 , 127159 166186 294310 136139 129129 147159 206210 150150 100100 140164 214222

AO3257 , 143167 166186 322326 139139 129129 147163 194194 134134 097097 144164 266266

AO3258 , 127143 166186 310310 139145 129129 147147 202206 134134 106118 144164 206214

AO3259 , 143159 166190 310326 139142 129129 147151 194202 134134 100118 136144 206266

AO3260 , 143163 186186 274346 139139 129129 143151 198214 134134 097097 140164 234250

AO3261 , 143143 186186 310326 139142 129129 151155 194206 134134 097097 144156 190190

AO3262 , 159167 186186 326346 139139 129129 147151 194202 134134 097097 144168 222266

AO3263 , 159167 186186 310310 136142 129129 147147 206206 134150 100118 144164 210210

AO3264 , 127159 186190 310326 139142 129129 143151 194194 134150 097127 144148 266266

AO3265 , 127143 166186 294358 136139 129129 143151 210218 150170 100103 140144 218222

AO3266 , 163171 186186 314346 139139 129129 147151 206218 134150 100100 156168 206214

AO3267 , 127159 166186 310326 136139 129129 147147 206210 150170 100106 144164 190222

AO3268 , 127127 166186 294326 130142 129129 147147 210214 150170 100118 140144 222250

AO3269 , 127143 186190 310310 142142 129129 147151 194206 134150 097100 144148 190190

AO3270 , 143143 186186 310326 136142 129135 151151 194202 134134 097100 144164 202266

AO3271 , 143159 186186 310326 136139 129129 143147 206206 134134 097097 144164 190210

AO3272 , 127127 166186 310358 136142 129129 147163 206218 134134 097106 144144 214218

AO3273 , 167171 186186 310346 139142 129129 143155 202202 150170 097100 140144 190206

AO3274 , 159183 186186 274310 127136 129129 131147 202206 134153 097097 164164 210214

AO4062 , 127163 166166 310322 139139 129129 147151 202214 134134 097118 136140 214214

AO4063 , 143167 186186 310314 142142 129129 151151 194206 150170 100100 144144 202266

AO4064 , 127167 166186 298326 121136 129129 147147 206206 134150 100106 140144 190210

AO4065 , 127143 186190 310326 139142 129129 147147 202202 134134 097097 144148 190206

AO4066 , 127143 166186 310322 139142 129129 147147 202206 134134 097097 136144 190190

AO4067 , 143187 186186 310322 130139 129129 151155 202210 134134 106115 148164 230270

AO4068 , 127143 186190 310326 130139 129129 147147 202214 134134 097100 136144 206214

AO4069 , 143143 186186 314322 139142 129129 147151 194206 150150 097100 144164 198266

AO4070 , 127143 166186 298322 139142 129129 147147 202202 134134 097100 144148 190222

AO4071 , 143143 186186 294326 133142 129129 147155 202206 150170 097118 144152 190222

AO4072 , 143143 186186 298346 139142 129129 139147 202202 134150 097097 144168 222234

AO4073 , 143167 166186 310310 121142 129129 143155 202202 134134 100118 144148 206206

AO4074 , 127159 166186 274314 139139 129129 147155 202230 134153 100103 148156 190226

AO4075 , 127143 186186 310310 139139 129129 143151 210214 134150 100115 156164 230250

AO4076 , 143171 186186 298310 136142 129129 147151 198202 134134 094118 144148 222234

AO4077 , 163167 186186 310326 139142 129129 155163 194202 134150 097118 140144 214266

AO4078 , 143183 166186 318346 127139 129129 135147 202206 134153 097118 144160 214214

AO4079 , 127143 186186 310318 127139 129129 135147 202206 134134 097118 144144 214214

AO4080 , 143163 166186 310326 130139 129129 147151 202202 134134 094118 144160 214222

AO4081 , 127143 186190 274310 139139 129129 143147 202206 134134 097097 148148 190206

AO4082 , 143143 186186 298310 142142 129129 147147 194202 134134 097118 144144 222266

AO4083 , 127171 186190 322322 136139 129129 147147 202206 134134 097100 136164 190210

AO4084 , 143187 186186 278326 136142 129129 151155 206206 150170 097100 144144 198210

AO4085 , 143171 166186 298310 130139 129129 147163 202202 134134 097097 144160 206218

AO4086 , 143183 186186 318358 142145 129129 147151 198202 153170 097118 144156 210226

AO4087 , 127127 186190 322322 127139 129129 147155 194214 134134 097097 136136 214266

AO4088 , 143183 186186 310314 139139 129129 147155 202206 134170 097100 144156 190214

AO4089 , 127175 166186 310318 127139 129129 143147 202206 134153 100103 144144 214214

AO4090 , 127175 166186 310318 127139 129129 143147 202206 134150 100103 144144 214214

AO4091 , 143183 166186 310318 139139 129129 135147 202206 134134 103118 144144 190214

AO4092 , 143167 186186 322326 136142 129129 147147 194202 134134 097097 144156 222266

AO4093 , 143159 186186 310310 130139 129129 147155 206214 134134 097115 140144 210226

AO4094 , 143155 186190 310310 130142 129129 147147 190198 150170 097097 144148 266266

AO4095 , 127167 166186 274294 133139 129129 147151 202202 150170 097100 136156 202234

AO4096 , 127163 166186 314322 127139 129129 147159 206214 134150 097100 136140 210214

AO4097 , 127143 186190 322326 139142 129129 147147 202202 134134 097097 136144 190222

AO4098 , 127143 186186 310310 130139 129129 147147 202206 134170 097106 144164 206206

AO4099 , 143163 186186 310326 139139 129129 147151 206206 134134 118118 144144 190210

AO4100 , 127159 186186 310314 127139 129129 143147 143147 134134 097103 144156 190270

AO4101 , 143167 166186 310314 136139 129129 147151 194206 134150 100100 144160 202266

AO4102 , 143163 166186 326358 130145 129129 147151 198210 134170 097118 144156 230234

AO4103 , 127187 186190 310326 142142 120129 151155 206206 134150 100118 144144 186190

AO4104 , 143143 186190 310310 142142 129129 143155 202202 134134 100118 144148 206206

AO4105 , 127183 186186 310314 139148 129129 147147 202210 150153 097103 156164 190222

AO4106 , 127143 166186 274322 130139 129129 139147 202214 134134 097118 136168 206214

AO4107 , 143143 186186 310346 136142 129129 151155 194198 134134 097100 144168 234266

AO4108 , 175183 166186 314346 127139 129129 143147 202206 134150 100103 156160 190214

AO4109 , 127143 166186 294322 130139 129129 147151 202214 134150 094097 136152 214218

AO4110 , 127175 186186 310318 139139 129129 143147 202206 134153 103118 144156 190214

pop

AO4868 , 143167 186186 310322 115115 129129 135143 202206 134150 097100 140156 238270

AO4869 , 143155 166186 318346 127130 129129 139147 202202 134150 100118 140148 190198

AO4870 , 155167 186186 274314 124139 129129 139163 202226 150153 118118 156160 198238

AO4871 , 127167 186186 310330 124148 129129 151155 214218 134134 097118 136160 202202

AO4872 , 127142 186190 330350 155171 129129 143155 202206 134134 097118 144164 198270

AO4873 , 127171 186186 274350 124124 129129 151163 202214 134134 100118 144156 198214

AO4874 , 155155 186186 274330 124130 129129 147163 202202 134150 100115 144160 198226

AO4875 , 167171 186186 318334 121136 129129 151155 214218 153153 097103 152172 246246

AO4876 , 171175 186186 310346 136142 129129 143163 206222 134153 097112 140164 210254

AO4877 , 143155 186186 318350 124124 129129 139147 202218 150150 100115 148160 198202

AO4878 , 127175 186186 314322 124130 129129 163163 198218 134153 112118 160164 202254

AO4879 , 143183 186186 318318 115124 129129 147163 202202 134134 097118 144156 198214

AO4880 , 127155 186186 318350 124136 129129 147151 194202 134150 100100 148164 198238

AO4881 , 155171 186186 274326 124148 117129 143147 202202 134150 118118 144160 206270

AO4882 , 127159 186186 274274 127145 129129 131163 202206 150153 097097 144148 210242

AO4883 , 127167 186186 310314 145148 129129 131163 202202 150150 097103 144164 210234

AO4884 , 143155 186186 274274 124124 129129 143147 202218 134150 097118 148160 198202

AO4885 , 163171 186186 314334 118124 129129 147151 206218 134150 097103 160164 186242

AO4886 , 155155 186186 274338 127127 117129 139143 202202 134150 100115 156160 202270

AO4887 , 155155 186186 274350 124127 129129 139147 202218 150150 097118 160160 202270

AO4889 , 155175 186186 274314 124127 129129 139163 202206 134150 097118 156160 222262

AO4890 , 171187 186186 274338 124142 129129 147163 202206 134150 097118 144160 198210

AO4891 , 163163 186186 290330 142151 117135 147171 194206 134150 097118 144160 214238

AO4892 , 171171 186186 322322 136136 129129 151155 202210 150153 112118 152156 242250

AO4893 , 163179 186186 322326 118127 129129 151155 202230 150150 091091 144160 186234

AO4894 , 155159 186186 290310 127133 129129 155167 206218 134150 094100 148160 202214

AO4895 , 127155 166186 290314 130148 117129 147147 198206 134134 115118 152160 198202

AO4896 , 143155 186186 290334 118145 129129 151171 206206 134134 097118 140156 214214

AO4897 , 143155 186186 274290 118127 129129 139151 202206 134150 100100 156160 214270

AO4898 , 171179 186186 318350 139151 129129 151163 202202 134134 100118 156164 210226

AO4899 , 127155 186186 310310 139139 129135 147159 202206 134134 097097 164164 198202

AO4900 , 155175 186186 274310 127139 129129 147167 202202 134150 097100 136160 274298

AO4901 , 155171 186186 290330 142145 129129 143151 206206 134150 094097 144160 198214

AO4902 , 175183 186186 274318 124130 129129 147163 202218 134134 097118 144144 202210

AO4903 , 155171 186186 310318 127130 129129 147147 194202 134150 118118 136148 194270

AO4904 , 171187 186186 330334 121127 129129 139143 202206 134150 097103 160160 210214

AO4906 , 163171 186186 314314 133145 129129 147151 194218 134150 103103 144156 238238

AO4908 , 127171 186186 274314 124124 129129 147147 202202 134134 100118 144156 210214

AO4909 , 143171 186186 314350 127148 129129 147155 202206 134134 094097 144164 202214

AO4910 , 179183 186186 274314 124139 129129 151163 202202 134134 097118 144156 210214

AO4911 , 155159 186186 314314 124133 129129 151167 206218 134150 100103 144148 202246

AO4912 , 163163 186186 290350 118127 129135 151151 194218 134150 094118 156164 230238

AO4913 , 143159 186186 314326 136145 129129 147151 206226 134134 103118 144172 214238

AO4914 , 155179 186186 310318 127127 129129 147147 198202 134150 100100 136148 202202

AO4915 , 143155 166186 318346 127148 129129 147163 202206 134150 097118 148152 198234

AO4916 , 171179 186186 274314 124124 129129 147163 202202 134134 097118 144164 210214

AO4917 , 155187 186186 274338 127142 129129 139147 202206 150150 097118 156160 198270

AO4918 , 127143 186186 294318 130130 129129 151155 202226 150150 097118 140144 202214

AO4919 , 127171 186186 274274 124136 129129 147151 202202 134134 097100 144164 210238

AO4920 , 143155 186186 274298 127127 129129 139159 202218 134150 097100 156160 202270

AO4921 , 127155 186186 274350 127136 129129 139151 194202 134150 100118 160164 238270

AO4922 , 143155 186186 274346 124130 129129 147163 202202 134150 118118 152160 190270

AO4923 , 163163 186186 290330 127151 117135 147151 194218 150150 094118 144160 230238

AO4925 , 155171 186186 318318 115139 129129 163167 202218 134134 097097 144156 202210

AO4926 , 143159 186186 314330 115145 129129 151155 206206 150153 097103 144160 214214

AO4927 , 143155 186186 318346 124124 129129 147171 202202 134150 097118 148152 210270

AO4928 , 179183 186186 318350 124136 129129 147151 194214 134134 100118 156164 198238

AO4929 , 143183 186186 274310 130139 129129 151155 194218 134150 121133 144144 194202

AO1322 , 143167 186186 310310 127139 117129 135151 194194 134134 100118 136140 194246

AO1323 , 143171 186186 338350 124139 129129 135155 194218 134134 097103 160160 202246

AO1324 , 155163 186186 314318 124139 129129 139147 202202 134150 097118 140148 198226

AO1325 , 163163 186186 290290 142151 129135 151171 194218 150150 097118 144156 230238

AO1326 , 143143 186186 310338 130142 129129 147151 194202 134134 097100 140140 194194

AO1327 , 171179 186186 310330 124136 129129 163167 218222 134153 100118 160164 202254

AO1328 , 127143 186186 314318 139142 129129 147151 194194 134134 097118 144144 226238

AO1329 , 155163 186186 274350 127130 129129 147151 202218 150150 118118 148160 198226

AO1330 , 143159 186186 290290 118127 129134 147151 206218 150150 097118 144156 222230

AO1331 , 171179 186186 274318 124124 129129 147147 194202 134134 100118 144156 210238

AO1332 , 143143 186186 314338 139151 129129 135163 194206 134134 094103 156160 214246

AO1333 , 143155 186186 318338 127130 129129 147147 202214 150150 100118 148156 198230

AO1334 , 171187 186190 310314 136142 129129 163163 194206 134134 103115 136164 194198

AO1335 , 143155 186186 310338 139142 129129 135147 202206 134150 097103 140144 214270

AO1336 , 155167 186186 318334 124130 129129 139171 202218 150150 100100 148152 198250

AO1337 , 155175 186186 322326 136139 117129 143151 202222 134153 100118 164172 206254

AO1338 , 155163 186186 274318 118124 129129 139143 202218 134150 097118 148148 202270

AO1339 , 155163 186186 274274 118124 129129 143147 202218 134150 097100 136148 202270

AO1340 , 143175 186186 290310 151151 129129 151159 218218 134134 097127 156156 226230

AO1341 , 159163 186186 290290 118127 129135 147151 206218 134150 094118 144156 222230

AO1342 , 155175 186186 274314 100127 129129 147163 202218 134150 097100 160160 198202

AO1343 , 155187 186186 298318 124127 129129 143147 202206 134150 097100 148156 214270

AO1344 , 159171 186186 274338 124133 129129 147151 202214 134150 100118 144156 198226

AO1345 , 127127 186186 310318 124139 129129 147163 202218 134134 097118 136156 202210

AO2043 , 155167 186186 318322 124130 129129 147163 202226 150150 094118 140148 198238

AO2044 , 163163 186186 330350 142151 129135 151171 194218 134150 097118 160164 230238

AO2045 , 155159 186186 318346 127142 117129 147151 198202 134150 100118 144148 198202

AO2046 , 143143 186186 310350 115124 129129 147151 202206 134150 097100 140160 214270

AO2047 , 143155 166186 318346 127148 129129 147147 202206 134150 118118 148152 234270

AO2048 , 143183 186186 274318 115124 129129 163163 202202 134134 097097 144144 210214

AO2049 , 127171 186186 274350 124136 129129 147151 194214 134134 097100 144164 198238

AO2050 , 143143 186186 310310 115115 129129 151167 194202 134134 097100 136140 202246

AO2051 , 179183 186186 274314 124136 129129 147147 194202 134134 118118 144164 210238

AO2052 , 143187 186186 338338 139151 129129 135147 202218 134134 097100 156160 202270

AO2053 , 127175 186186 290314 124127 129129 139163 202206 134134 094100 156160 214214

AO2054 , 143143 166186 310346 130139 129129 147151 194202 134134 103118 140152 190246

AO2055 , 155171 186186 274274 124139 129129 147147 194202 134150 097118 144160 226270

AO2056 , 143159 186186 290350 142151 129135 151171 194206 134150 097118 144144 214238

AO2057 , 143155 186186 310338 130139 129129 143167 202202 134134 097097 140140 202202

AO2058 , 143163 186186 330350 142151 117135 147151 206206 134134 094118 144160 214222

AO2059 , 143183 186186 274314 124130 129129 147163 214218 134134 097118 144160 198202

AO2060 , 155155 186186 318338 127130 129129 147151 202202 150150 097100 148156 238298

AO2061 , 143175 186186 274330 118142 129129 151159 194218 134150 094115 140140 230242

AO2062 , 143143 186186 310346 115115 129129 151155 202202 150150 097103 000000 202270

AO2063 , 143143 186190 290310 118118 129129 151159 206218 134150 097127 144144 214226

AO2064 , 143163 186186 290334 118118 129129 147151 202218 134150 094118 000000 198230

AO2065 , 155179 186186 310318 124124 129129 139167 202218 134150 118118 136148 198202

AO2066 , 127155 186186 274346 139148 129135 151159 206210 134150 097115 144144 206230

AO2067 , 163179 186186 330330 124151 117129 155171 218218 134150 097097 140160 202230

AO2068 , 143155 186186 318350 124127 000000 147163 202202 150150 097100 136148 214226

AO2072 , 159187 186186 290310 139142 129135 147155 202206 134134 097118 144164 242242

AO2074 , 171175 186186 274322 124136 129129 147151 206218 134150 100118 148148 214238

AO2084 , 143143 186186 274330 151151 117129 151171 218218 134134 094097 156160 202230

AO2085 , 143179 186186 290330 118118 117129 155171 218218 134150 094097 156160 202230

AO2086 , 143167 186186 274338 124139 117129 151159 194202 134134 103118 148160 194270

AO2087 , 143171 186186 314318 115124 129129 147163 202214 134134 097097 156160 198214

AO2088 , 127163 186186 274290 118124 117129 159171 202218 134170 094097 156156 194230

AO2090 , 155171 186186 274274 124139 129129 147147 194202 134150 097118 144160 226270

pop

AO1267A, 127171 186186 290314 130133 129129 143151 214218 150153 109112 160160 190250

AO1268 , 163171 186186 322322 124133 129129 151155 214218 134150 109112 144144 190250

AO1269 , 167171 186186 306322 124127 129135 151171 214230 134134 097097 144156 250250

AO1270 , 127143 186186 290322 124133 129135 151155 214226 150153 097130 144160 214250

AO1271 , 127171 186186 290314 124133 129135 151155 218226 150153 112130 160160 214270

AO1272 , 127143 186186 290322 124133 129129 151155 218226 150153 097109 160160 214270

AO1273 , 175179 186186 282330 124139 129129 139151 210218 134134 091103 152156 218222

AO1274 , 167171 186186 290306 124127 129129 143151 218222 127153 097112 156160 226270

AO1275 , 143163 186186 314322 124133 129135 143151 214218 134150 097109 144160 190250

AO1276 , 163171 186186 306322 127133 129129 151171 214230 127153 097097 144156 250254

AO1277 , 127171 186186 290322 127133 129129 143151 218218 150153 109112 144160 190270

AO1278 , 143163 186186 290290 124127 129135 151171 218230 134153 097112 156160 250270

AO1279 , 143167 186186 290314 127127 117129 151159 210218 150153 097109 160160 218270

AO1280 , 143167 186190 290314 127127 129129 151151 210218 134153 097112 160160 218270

AO1281 , 127171 186186 322322 124127 129129 143151 214218 150153 112130 144144 190250

AO1282 , 163179 186186 282306 124124 129129 139143 210222 134134 097097 152156 218222

AO1283 , 127175 186186 282330 136139 129129 139151 202222 134134 097103 152156 242254

AO1284 , 143167 186186 290306 127133 129129 151171 214222 134134 097112 156160 226250

AO1285 , 143167 186186 290306 127127 129129 143151 218230 134153 097097 156160 250270

AO1286 , 143163 186186 306310 115124 129129 147151 202222 134134 097103 156160 222230

AO1287 , 163167 186186 290290 121127 129135 143151 218222 134153 097112 156160 226270

AO1288 , 163171 186186 314322 127133 129135 151155 214226 150153 097130 144160 214250

AO1289 , 143163 186186 314322 124127 117129 151151 214214 150153 097109 144160 226250

AO1290 , 127179 186186 322346 127145 129129 155167 202222 134134 097103 140160 230254

AO1291 , 167171 186190 314322 124127 129129 151151 214214 134150 097109 144160 226250

AO1292 , 167171 186186 290290 127133 129129 151171 214222 134153 097100 156160 226250

AO1293 , 127127 186186 310334 124124 129129 143147 226226 150150 097097 148148 218270

AO1294 , 163171 186186 322322 127127 129129 151155 218218 134150 109112 144144 000000

AO1295 , 143163 186186 290314 124127 129135 143151 218218 150153 097109 160160 190270

AO1296 , 127171 186186 290314 124133 129135 151155 214218 150153 097109 160160 190250

AO1297 , 167171 186186 290306 124127 129129 143151 218230 127134 097112 156160 250270

AO1050 , 175179 186186 282306 124139 129129 139151 202210 134134 097097 152156 218242

AO1052 , 163179 186186 282306 124124 129129 139151 222222 134134 097097 152156 222246

AO1053 , 163179 186186 282330 124124 129129 143147 202222 134134 097103 152156 242254

AO1054 , 163179 186186 282330 124124 129129 143147 202222 134134 097103 152156 242254

AO1055 , 175179 186186 306322 136139 129129 139143 222222 134134 091103 156168 222254

AO1056 , 175179 186186 282306 124124 129129 139143 210222 134134 097103 152156 218222

AO1058 , 143175 186186 310330 115124 129129 143147 202202 134153 097103 156160 230242

AO1059 , 143175 186186 310330 115139 129129 143147 202222 134134 091103 156160 222230

AO1061 , 127143 186186 322334 127133 129129 163167 222222 134134 091103 140148 214254

AO1062 , 171171 186186 310310 133142 117129 143151 226234 150150 097097 148160 214242

AO3873 , 171175 186186 330334 127130 129135 151155 202218 134153 109112 156172 242246

AO3874 , 143167 186186 274294 124127 000000 155167 202206 134134 097103 148160 214230

AO3875 , 127171 186186 298310 124133 129135 143167 210234 134150 097103 148148 214218

AO3876 , 143163 186186 290290 127136 129129 163171 202214 127153 097103 156168 230250

AO3877 , 143163 186186 290334 124136 129129 147163 202230 134153 097103 152168 214234

AO3878 , 163175 186186 290334 136136 129129 163163 202230 153153 094103 152168 214234

AO3879 , 127167 186186 314334 124127 129129 143163 190218 134153 100103 000000 214230

AO3880 , 143175 186186 310310 124127 129129 147155 214222 134134 094097 000000 222222

AO3881 , 127127 166186 314314 127142 129129 143143 190218 150153 097103 160164 190230

AO3882 , 167175 186186 290310 115127 129129 147147 202218 134153 097103 140160 214214

AO3883 , 167171 186190 330334 118121 000000 143163 214218 134153 103112 000000 246254

AO3884 , 143163 186186 290338 124136 129129 159163 202206 134153 100103 160168 206214

AO3885 , 143163 186186 310334 115124 129129 163163 202206 153153 094103 152160 230274

AO3886 , 163171 186186 322334 130136 129135 155163 214218 134134 103112 164172 214250

AO3887 , 143175 186186 310330 124136 129129 139147 202222 134153 103103 156160 214222

AO3888 , 163175 186186 290334 136136 129129 163163 202230 153153 094103 152168 214234

AO3889 , 171179 186186 282322 124133 129129 139151 222226 134150 091103 152152 218254

AO3890 , 167171 186186 310310 127136 129129 159167 214234 150150 103103 148152 194214

AO3891 , 143163 186186 290306 136136 129129 143163 202210 134134 097103 156168 218230

AO3892 , 127171 186186 310314 124124 129129 147163 206218 134150 100109 160160 206214

AO3893 , 143175 186186 310314 124127 129129 151155 202214 134153 094097 160160 222242

AO3894 , 175179 186186 282290 115136 129129 143147 202210 134134 097103 152160 214218

AO3895 , 127171 186186 290322 121133 129129 155163 206214 134153 094100 156160 206222

AO3896 , 163175 186186 310314 124127 129129 163167 202210 134134 091100 160160 214218

AO3897 , 163171 186186 290314 127133 129135 143167 222222 134150 091097 156160 226254

AO3898 , 127163 186186 314322 124124 129135 143167 222222 134150 097100 160164 226254

AO3899 , 179179 186186 282290 124124 129129 151167 210222 134134 097103 144152 218254

AO3900 , 127171 186186 310322 121124 129129 155163 214218 134134 094100 160164 214222

AO3901 , 127127 186186 290350 127127 129129 143163 218218 150153 097109 140156 190214

AO3902 , 127127 186186 322322 124133 129129 167167 210214 134150 097097 140152 194218

AO3916 , 163179 186186 290298 127133 129129 151171 214230 134153 097097 156156 226250

AO4030 , 175179 186186 306322 124124 129129 147151 222222 134134 097097 156168 222254

AO4031 , 143163 186186 290322 127133 129135 151171 214230 127153 097097 144156 250250

AO4032 , 127167 186186 310338 124142 129135 151159 214214 150150 097109 140160 226226

AO4741 , 127171 186186 310322 118127 129129 143143 206222 134134 097097 148152 222258

AO4742 , 171175 186186 290342 118124 129129 143143 206222 134153 103103 152168 226254

AO4743 , 143167 186186 314322 118127 129129 143143 190222 134150 097103 160160 186246

AO4744 , 127163 186190 282338 127136 117129 143143 214222 134150 091097 140152 222226

AO4745 , 127163 186190 314322 127130 129135 151163 214222 150153 097103 156164 194226

AO4746 , 155155 186186 314314 000000 000000 143155 230230 000000 103103 156160 254254

AO4747 , 127163 186186 282314 124127 129129 139155 218222 134150 091103 152160 194254

AO4748 , 127127 186186 298346 124127 117129 151159 218226 150153 097109 148164 214242

AO4749 , 163171 186186 314322 124127 129129 147147 202210 134134 097103 160160 214218

AO4750 , 127163 186186 290330 115124 129129 147151 202202 134134 103103 152160 230242

AO4751 , 127127 186186 290330 124139 129129 143159 202218 134153 097109 140156 214242

AO4752 , 127175 186186 298330 124139 129129 139159 218222 134134 097100 160164 214254

AO4753 , 163171 186186 326334 118124 129129 155155 214214 153153 094097 160160 214226

AO4754 , 163163 186186 290290 127127 117129 151159 214218 134134 097100 140140 214226

AO4755 , 127175 186186 306346 124142 129129 151159 218226 134134 097109 148156 194242

AO4756 , 127167 186186 290314 124130 129129 159159 218222 134134 100100 140160 194214

pop

AO0713 , 127143 166186 322342 130139 129129 143155 218218 153153 094103 160160 250278

AO0714 , 167167 186186 290318 121130 129129 143155 206226 134150 094118 160172 242250

AO0715 , 143171 166186 314318 124130 129129 143155 222230 134153 094118 160172 214254

AO0716 , 127179 166186 322322 121121 129129 151155 218234 134153 097097 152160 186242

AO0717 , 167175 166186 314318 130142 129129 155155 218218 150153 097097 152152 186186

AO0718 , 127167 186186 322342 118124 129129 131155 206214 134153 097103 160160 186266

AO0720 , 167179 186186 314318 121139 129135 155155 218218 134153 097118 152168 186246

AO0721 , 171171 186186 314346 121121 129129 143155 226230 134134 094097 156168 214246

AO0722 , 167167 186186 314322 142142 129129 155155 206218 134150 097118 152160 234250

AO0723 , 127127 186186 290314 142142 129129 155155 218218 150153 097136 152160 186230

AO0724 , 127171 186186 310326 121127 129129 151155 218218 150153 094097 156160 186234

AO0725 , 127167 186186 290322 127142 129129 155155 214230 134153 097097 160160 186246

AO0726 , 171171 186186 322346 121124 000000 155155 218226 134153 094097 152156 234242

AO0727 , 127175 186186 290314 121130 129129 151155 214218 134153 097136 152160 186246

AO0728 , 127143 186186 322342 124139 129129 155155 206226 150153 097118 152160 242250

AO0730 , 171171 186186 322322 121145 129129 151155 226230 134150 094097 152172 246250

AO0731 , 167171 186186 306322 121124 000000 131151 218234 000000 097097 156160 230242

AO0732 , 127175 186186 314322 130130 129129 143155 214214 150153 097118 152152 246278

AO0733 , 167167 186186 314322 121130 129129 155155 206218 150150 094097 152160 186250

AO0734 , 167171 186186 290314 121121 129129 151155 218234 150153 094097 160160 234266

AO0735 , 127127 166186 322322 130139 129135 155155 214218 153153 097097 152160 186246

AO0736 , 171175 186186 318322 121124 000000 143155 206226 134153 097118 160172 242258

AO0737 , 167171 186186 290326 121130 129129 155163 206218 134150 097118 152160 250250

AO0738 , 167171 186186 314314 121121 129129 155155 218234 134150 097118 160168 230266

AO0739 , 171171 186186 306322 121142 129135 143143 222226 134134 094100 152160 246258

AO0740 , 167171 186186 290310 130136 129135 155163 218218 134134 094094 156160 190234

AO0741 , 127143 186186 326346 124124 129129 143147 206214 134134 097118 152160 246250

AO0742 , 167167 186186 322342 118136 129135 143159 206214 150153 097097 152160 246258

AO0743 , 127175 186186 326326 115130 129129 151155 214230 150153 097103 148160 186218

AO0744 , 167171 186186 290322 121130 129129 151163 218234 134153 097118 160160 250266

AO0745 , 127171 186186 290326 121130 129135 151155 218234 150150 103118 152160 250266

AO0746 , 127179 186186 306322 124127 129129 155163 214218 134153 103118 152156 230246

AO0747 , 179183 186186 342342 127139 129129 155155 202218 134134 097118 156160 186238

AO0748 , 127175 186186 322322 121127 129129 143155 214218 153153 097097 140152 186226

AO0749 , 167171 166186 290318 130130 129129 143151 206222 150153 094118 160172 250254

AO0750 , 127171 186186 314318 121142 129129 151155 218234 134134 118118 152160 230266

AO0751 , 167171 186186 314322 130130 129129 143151 206222 134153 097097 156160 254258

AO0752 , 167179 186186 314342 124127 129129 155155 202218 134134 097118 160168 186238

AO0803 , 167167 186186 322342 118124 129135 143143 206206 150153 097118 152160 226258

AO0804 , 127179 186186 318322 127139 129129 155155 214218 134150 097097 152152 186246

AO0805 , 167171 186186 322346 121127 129135 155155 226226 134150 097097 156160 242246

AO0806 , 171171 186186 314322 121124 129129 155155 218226 134134 097097 152168 230246

AO0807 , 127171 186186 306346 127127 129129 143171 218218 134134 097097 156160 234246

AO0808 , 171171 186186 310342 121130 129129 155163 206218 134153 103118 160160 250250

AO0809 , 167171 186186 322322 124127 135135 143159 206206 150153 097097 152152 250258

AO0810 , 171175 166186 310314 121130 129129 155155 214214 150153 097118 152160 246270

AO0811 , 127167 186186 290290 127142 129129 155155 206214 134153 097097 160160 186246

AO0812 , 127127 186186 290322 130139 129129 143155 218218 134153 094097 152160 186234

H 232 , 167179 186186 322322 124127 000000 143155 214218 134134 097103 152168 198226

H 238 , 000000 186186 322322 121124 000000 151155 206214 000000 097097 156160 242246

H 488 , 127127 186186 322322 127136 129129 155163 214218 150153 097097 152160 246250

H 489 , 167167 186186 000000 121124 000000 131163 218218 134134 097097 160160 190190

H 498 , 167171 186186 000000 118124 129129 155171 218218 134150 097109 000000 198214

AO4540 , 127167 166186 314322 121136 129129 151151 214234 150153 097097 152160 198266

AO4541 , 127127 166186 322322 127127 129135 143155 214230 150153 097118 152160 214246

AO4542 , 127143 166186 290322 121139 129129 151155 206218 134150 097118 160160 186250

AO4543 , 167171 186186 306338 115136 129129 155163 206218 153153 094097 152160 222226

AO4544 , 127179 186186 318322 124130 129135 155155 218218 150153 094118 152152 246246

AO4545 , 127127 186186 290322 121121 129129 151151 214214 150153 097097 160160 198198

AO4546 , 167175 186186 290322 121124 129135 143155 214214 134153 094097 160160 246246

AO4547 , 127171 186186 322322 121124 129135 143163 206218 150153 097118 140160 230250

AO4548 , 167167 166186 310322 127139 129129 155155 218218 134153 097103 152160 230234

AO4549 , 127167 186186 314322 121124 129129 155167 206214 134150 097097 160168 230250

AO4550 , 167171 186186 310322 118124 129129 143155 218230 134150 097118 152156 186214

AO4551 , 127127 186186 322326 121124 129129 151151 214218 134134 097097 152160 186230

AO4552 , 167167 186186 290326 130136 129135 151151 206214 150150 097103 160160 198250

AO4553 , 143171 186186 322326 127139 129129 143163 226234 134150 097100 160164 226246

AO4554 , 167171 186186 310342 127142 129129 131143 234234 134150 097103 156160 230278

AO4555 , 167171 186186 290342 127127 129129 155163 214218 134153 097118 156160 246278

AO4556 , 127171 186186 322346 121124 129129 147155 206214 134150 097118 152156 246250

AO4557 , 143163 186186 310346 121124 129135 151155 226230 153153 097097 156160 214242

AO4558 , 167167 186186 322342 127130 129129 143155 206218 150153 097097 156160 186226

AO4559 , 171179 166186 322322 121127 129135 155163 214218 134150 097118 152160 234246

AO4560 , 127179 186186 322342 127142 129129 147155 218218 134150 097097 152160 186186

AO4561 , 127167 166186 322322 121124 135135 143151 206234 134153 097103 156160 234242

AO4562 , 127163 186186 322322 121130 129135 143155 214218 150153 094097 160160 234246

AO4563 , 175179 186186 322322 124127 129135 155155 214218 134153 097118 152152 186246

AO4564 , 171175 186186 318322 124142 129129 147159 214230 134150 118118 152160 198214

AO4565 , 143171 186186 290322 124139 129129 143155 218234 134134 094118 152160 226234

AO4566 , 127175 186186 322322 124139 129129 143155 214218 150153 097097 152160 198234

AO4567 , 127167 186186 322346 115127 129129 147155 218222 134153 097097 152156 186254

AO4568 , 127127 186186 314322 121121 129129 143155 218230 134150 097097 152168 186214

AO4569 , 167179 186186 322322 121139 129129 151155 214214 127150 097103 160160 198246

AO4570 , 127127 186186 322322 127139 129135 155155 214214 150153 097097 152160 230246

AO4571 , 127171 186186 306326 121145 129135 151159 214214 150153 097097 152160 186198

AO4572 , 127163 186186 314322 115124 129135 143155 218218 134150 094118 152160 190246

AO4573 , 127163 186186 310342 121127 129129 131155 214218 134153 097118 160160 230230

AO4574 , 171171 186186 322322 124130 129129 143155 214226 134153 097097 152160 230242

AO4575 , 171175 186186 322342 124139 129129 143151 218218 134153 097097 156160 186234

AO4576 , 127171 186186 322322 121124 129135 143163 206218 150153 097118 140160 230250

AO4577 , 163167 186186 310314 127127 129129 143143 230230 150150 097097 160160 214286

AO4578 , 171175 186186 306342 124142 129135 143155 218222 000000 100103 156160 198258

AO4579 , 167167 166186 322342 124130 129129 155167 202214 134153 094097 156156 238270

AO4580 , 127167 186186 290338 124127 129135 151155 230234 153153 097118 152160 214266

AO4581 , 127127 186186 314322 118142 129135 155155 218218 134134 100103 152160 190230

AO4582 , 127171 186186 290314 127142 129129 131167 214218 134150 097118 160160 186246

AO4583 , 167175 186186 290322 121139 129135 143151 214218 153153 097103 152160 190198

AO4584 , 127167 186186 314326 121130 129135 143155 214218 150153 097103 152152 190246

AO4585 , 127171 186186 314318 124130 129129 143151 218230 134150 097118 152168 214230

AO4586 , 127167 186186 290322 121139 135135 143155 214214 150153 097103 160160 246246

AO4587 , 127175 166186 342342 121124 129129 143155 206214 150153 097097 160160 226230

AO4588 , 167171 186186 318338 127130 129135 143155 218234 150153 097097 152160 226230

AO4589 , 127167 186186 322322 124127 129135 131155 218218 134153 097103 160160 186230

AO4590 , 143171 186186 314318 124130 129135 143155 222230 150153 094097 148160 214254

AO4591 , 127167 186186 338342 121130 129129 147155 218234 000000 097097 152152 246246

pop

AO4638 , 171171 186186 290322 124145 129135 143163 214218 153153 097097 160164 242242

AO4639 , 179179 186186 286314 127127 129135 143147 202226 134150 097100 160160 206234

AO4640 , 171171 186186 318334 124142 135135 151155 214218 150153 094097 152164 186246

AO4641 , 143171 186186 290334 124127 129129 143143 210226 134153 094097 152156 250258

AO4642 , 127171 186186 322346 127133 129135 143155 202222 150153 094097 160164 242246

AO4643 , 127167 186186 290322 115130 129129 143155 206214 150153 097103 152152 242250

AO4644 , 143163 186186 322346 127136 129135 151151 218218 134153 109133 140152 246246

AO4645 , 171171 186186 326334 127133 135135 155163 206222 150153 103136 152156 186242

AO4646 , 143171 186186 318322 121127 129129 151163 214222 134153 118133 140140 238250

AO4647 , 127171 186186 290326 136148 129129 151155 222222 134153 112112 156160 230238

AO4648 , 127167 186186 326342 127130 129129 143155 214222 134153 094094 152164 258274

AO4649 , 143167 186186 290346 124136 129129 143163 194214 134153 094103 140152 238238

AO4650 , 127159 186186 306318 121136 129129 143151 214214 134153 106112 148156 214238

AO4651 , 171171 186186 290342 127136 129129 151155 210210 150153 118133 160160 186238

AO4652 , 143171 186186 342346 115121 129135 143155 202206 134150 094103 152156 222238

AO4653 , 127171 186186 294346 121148 129135 163167 210214 150150 094097 156160 218246

AO4654 , 171171 186186 294334 142145 129129 151155 206230 134150 094097 164164 222238

AO4655 , 163163 186186 318322 118124 129135 131151 202214 150150 103136 152156 246250

AO4656 , 127167 186186 290350 127142 129129 151155 210214 134150 097115 152160 230238

AO4657 , 143171 186186 306322 133136 129129 151163 214226 134150 097118 152156 226246

AO4658 , 171171 186186 322346 121124 129129 151151 226226 150153 109112 160160 250250

AO4659 , 127171 186186 322342 121142 129129 151163 214226 150153 097103 160172 242266

AO4660 , 127171 186186 294342 136142 129129 151155 206206 134150 097133 156160 238250

AO4661 , 167191 186186 306342 115136 135135 147151 214214 150153 112136 140144 238250

AO4662 , 127167 186186 306346 124133 129129 147155 210222 150153 112112 156160 218254

AO4663 , 143143 186186 306318 127136 135135 151151 206218 134153 097109 140156 230242

AO4664 , 127171 186186 330342 124127 135135 151155 222234 153153 097103 152160 226270

AO4665 , 127171 186186 306342 124130 129129 155155 214222 134134 097097 156172 238242

AO4666 , 127171 186186 326334 121142 129129 155163 202214 134150 097115 152160 226254

AO4667 , 127167 186186 326338 118121 129135 155155 210222 150153 097118 164168 238246

AO4668 , 127171 186186 322342 127136 129129 151151 222234 134153 097133 160164 230238

AO4669 , 167171 186186 342342 121121 129129 151151 210226 134153 097118 160160 250266

AO4670 , 143167 186186 322342 127142 129129 143151 206206 134150 097118 140152 238254

AO4671 , 163167 186186 290318 115124 129129 155159 214214 150153 097112 152160 238250

AO4672 , 167171 186186 322334 124130 135135 151155 202218 153153 094097 152156 238246

AO4673 , 171179 186186 290322 121124 129135 155155 202218 153153 094115 156160 226246

AO4674 , 127127 186186 306318 124124 129129 151163 214230 153153 115133 152156 214238

AO4675 , 127167 186186 322346 127136 129129 151155 214222 134134 097112 140152 238250

AO4676 , 171191 186186 322346 115127 129129 151155 206210 153153 097118 140164 238254

AO4677 , 143171 186186 322326 133136 127127 151163 206218 134153 097112 152186 186246

AO4678 , 167167 186186 306318 115127 129135 151155 202218 150150 103112 140152 226258

AO4679 , 127133 186186 290326 127133 129135 151155 202218 153153 094097 152156 246274

AO4680 , 163171 186186 322346 127127 129135 155163 206230 134153 094097 148160 222254

AO4681 , 163167 186186 334346 124124 129129 151155 226230 153153 097136 152160 222258

AO4682 , 167171 186186 322342 127142 129129 143151 222230 134153 103103 164164 222270

AO4683 , 163171 186186 334346 121148 129129 155167 202214 150153 097097 156160 238274

AO4684 , 167171 166186 322322 118142 129129 155155 206222 150153 097097 152160 186238

AO4685 , 163171 186186 318326 124133 129129 151151 218218 134153 097109 140152 246246

AO4686 , 167171 186186 322326 127133 129129 155155 202218 134150 097133 152156 238246

AO4687 , 127143 186186 334342 127130 129135 155163 222222 134153 097097 152160 242270

AO4460 , 163171 186186 322350 127142 129129 143155 214234 134134 097097 152164 238266

AO4461 , 143167 186186 290318 124148 129129 143155 202230 134134 103112 156160 186238

AO4462 , 163171 186186 294330 127142 129129 151167 214222 134150 097097 160164 238238

AO4463 , 127127 186186 294322 127142 129129 147155 222222 134150 097097 164164 238238

AO4464 , 167171 186186 330342 127136 129129 155155 210222 150153 112133 144156 242242

AO4465 , 167179 186186 310322 121127 129129 151163 214222 134134 097109 152164 242262

AO4466 , 171171 186186 318346 124124 129129 151155 218230 150153 097097 152164 194242

AO4467 , 127171 186186 322334 127145 129135 151155 206226 150153 094097 152160 238242

AO4468 , 127183 186186 290314 133136 129129 147155 214226 134150 097133 140152 234262

AO4469 , 127191 166186 322322 133133 129129 143155 202230 134134 097118 140164 254274

AO4470 , 171171 186186 290342 121124 129129 143155 206214 134150 097097 152152 190190

AO4471 , 171171 186186 322322 127136 129129 155159 214222 134153 097118 160164 242250

AO4472 , 167171 186186 318342 124136 129129 143155 218222 150153 094136 160164 242242

AO4473 , 167191 186186 322322 136151 129129 151155 214214 134150 097136 152164 234250

AO4474 , 143167 186186 346346 136139 129129 143155 202226 150153 094094 140160 238238

AO4475 , 171171 186186 322334 130142 129135 155155 202214 150150 094118 164168 226254

AO4476 , 171171 186186 322334 139151 129129 147155 214222 150153 094097 156160 194242

AO4477 , 143143 186186 290334 121124 129129 151155 214230 153153 097097 152160 222238

AO4478 , 127163 186186 294322 124142 129129 155167 214222 150153 097097 164164 238238

AO4479 , 127171 186186 330346 124124 129129 155167 214222 134134 097097 152160 238238

AO4480 , 127127 186186 306334 127127 129129 143163 222222 134153 112133 156168 238242

AO4481 , 127163 186186 318330 136148 129135 151155 206214 150153 094097 160160 214246

AO4482 , 171171 186186 290322 127145 129129 147155 214230 153153 112130 160164 226262

AO4483 , 127127 186186 322346 124124 129129 155167 214234 134134 097097 152164 238266

AO4484 , 143179 186186 318322 121121 129135 143151 214226 134153 097097 160160 238250

AO4485 , 167171 186186 322322 121142 129129 143155 202218 134153 094112 152160 238250

AO4486 , 167171 186186 322330 124139 129129 151167 214218 134134 097097 140160 214250

AO4487 , 127167 186186 322334 124136 129135 155163 210230 150153 094097 160160 222242

AO4488 , 163167 186186 342342 124124 129129 143151 214222 150153 103118 160160 230238

AO4489 , 127191 186186 290346 115136 129129 155155 206206 134150 097136 140160 186250

AO4490 , 163191 186186 346346 136142 129135 131155 222226 134134 103133 140164 226242

AO4491 , 171171 186186 322334 127145 129129 147155 206230 150150 103112 160164 186226

AO4492 , 127167 186186 306334 127127 129129 163167 214230 134150 094118 156160 190222

AO4493 , 143191 186186 334346 115142 135135 143151 206234 150150 097112 152164 226230

AO4494 , 127171 186186 290338 121124 129135 151151 206206 134153 097097 152164 190194

AO4495 , 163171 186186 290322 115142 129129 143155 206234 150150 112118 160164 226258

AO4496 , 127171 186186 294330 127142 129129 151167 222234 134153 097118 164164 238266

AO4497 , 143171 186186 330346 121136 129129 155159 222230 153153 097133 160160 222238

AO4498 , 163167 186186 290334 124124 129129 151155 214234 150153 097136 152168 238266

AO4499 , 175179 186186 290342 000000 129129 151163 206214 134153 109133 152160 262262

AO4500 , 171171 186186 322334 121121 129129 143155 218218 134153 097097 140160 242262

AO4501 , 171179 186186 290322 121148 129135 151163 214214 153153 094097 160160 190262

AO4502 , 143167 186186 290342 136148 129129 151163 206214 153153 103112 144160 246258

AO4503 , 143167 186186 322346 121127 129129 143155 206218 134134 097133 156168 250258

AO4504 , 143171 186186 290342 124136 129129 143159 218222 153153 097118 152160 238270

AO4505 , 171171 186186 322322 130130 129129 151155 218222 134153 097097 140160 242250

AO4506 , 127163 186186 294322 124127 129129 151167 222222 134153 097097 164164 238238

AO4507 , 127127 186186 342346 121124 129135 143155 210222 134153 097097 152156 238246

AO4508 , 143171 186186 322326 124127 129129 155155 202226 134153 097097 152160 238238

AO4509 , 167167 186186 322346 124127 129129 155155 214222 150153 112133 140164 238250

pop

AO4688 , 127167 186186 286298 121127 129129 147159 202202 150150 091091 160160 218250

AO4689 , 167167 186186 294294 136136 129129 139151 214214 150150 091109 164164 246246

AO4690 , 127167 186186 298306 118136 129129 151155 202214 150153 109118 148160 186214

AO4691 , 127127 186186 274294 127133 129129 147151 202202 134150 097097 148164 218226

AO4692 , 127127 186186 294310 124127 129129 151155 202210 134150 091094 156156 206226

AO4693 , 127127 186186 306314 127127 129129 147147 202210 150153 094097 148160 206234

AO4694 , 147199 186186 298310 124148 129129 151151 202226 134153 091097 160160 214218

AO4695 , 127159 166186 274318 133148 129129 151151 202210 150150 097097 148156 234250

AO4696 , 127163 186186 306310 115124 129129 147151 202214 134150 091118 148156 206226

AO4697 , 127127 186186 286294 127148 129129 151159 202210 150150 097097 156164 250250

AO4698 , 127167 186186 306330 124136 129129 147151 202214 150153 097118 148156 234246

AO4699 , 163167 186186 306326 133136 129129 151151 202202 150150 097118 148156 206226

AO4700 , 163167 186186 294326 130133 129129 147163 202234 150153 097118 156156 206226

AO4701 , 127163 186186 326326 124133 129129 151151 214214 150150 091097 152156 210246

AO4702 , 159163 166186 274290 115133 129129 151151 202210 134134 097097 148160 214226

AO4703 , 127167 186186 286294 127127 129129 159159 202202 150150 091109 160164 214218

AO4704 , 127127 186186 298346 124130 129129 151155 202214 150150 097097 148160 218254

AO4705 , 127163 166186 290318 127133 129129 151151 202210 134150 097097 160164 214234

AO4706 , 143199 186186 330334 130136 129129 147147 214222 150150 097109 148156 194246

AO4707 , 127127 186186 318334 130133 129129 147163 202202 134150 109115 156156 206218

AO4708 , 127127 186186 286294 118121 129129 151155 202214 150153 091097 160164 218246

AO4709 , 127163 186186 294310 115124 129129 147151 202210 134153 091118 156156 206226

AO4710 , 127127 186186 290330 127130 129129 147147 214222 150150 097109 148156 194246

AO4711 , 127167 186186 306334 124136 129129 147151 202210 150153 094109 148156 206218

AO4712 , 127127 186186 318318 127133 129129 151155 202222 150150 097103 156164 206234

AO4713 , 163167 186186 294294 124133 117129 147147 202202 153153 094097 156164 214226

AO4714 , 127199 186186 294330 124124 129129 147155 202210 150153 091094 156156 206214

AO4715 , 159167 186186 318330 133136 129129 147155 202202 134150 097109 156164 214226

AO4716 , 127167 186186 286326 127133 129129 155159 206222 150150 091091 152156 206250

AO4717 , 167199 186186 298326 124136 129129 151151 202202 150153 091097 156160 206218

AO4718 , 167199 186186 298326 118124 129129 147155 202214 150150 097097 160160 214246

AO4719 , 127127 186186 286334 127133 129129 147159 202202 150150 097097 152160 218234

AO4720 , 127163 186186 310326 121133 129129 151151 206214 150153 097097 160160 210222

AO4721 , 159167 186186 274326 133136 129129 147151 202202 150150 097097 148160 214226

AO4722 , 159167 186186 298318 118127 129129 147155 202214 150153 091091 156160 186214

AO4723 , 159167 186186 310326 130133 129129 155155 214214 134153 091097 156156 186210

AO4724 , 127167 186186 286294 127136 129129 151159 202202 150150 097097 160164 214250

AO4725 , 127127 186190 294306 127133 129129 147155 202210 134150 094103 156156 206226

AO4726 , 127167 186186 286330 121127 129129 151159 202202 150150 091097 156160 214218

AO4727 , 163167 186186 326346 130133 117129 151163 210218 150150 097097 148156 238246

AO4728 , 127163 186186 294306 115124 129129 151151 202210 134150 091094 152156 206226

AO4729 , 163167 186186 274326 115124 129129 151151 202202 153153 091097 160160 210218

AO4730 , 127163 186186 326330 124133 129129 155159 214218 150150 091097 156156 238246

AO4731 , 127167 186186 306330 118148 129129 147147 214214 150153 097118 148156 186246

AO4732 , 127167 186186 326326 118133 129129 151155 214214 134153 091097 156156 210210

AO3801 , 167167 186186 286318 130136 129129 155159 214222 150150 091097 156172 206218

AO3802 , 127199 186186 298326 115118 129129 151155 202230 134153 091097 160160 186218

AO3803 , 127199 186186 294298 118124 129129 147151 202214 150153 091097 160160 218246

AO3804 , 127127 186190 326334 115133 129129 147151 210214 150153 097109 140152 234238

AO3805 , 127179 186190 294334 115124 129135 147151 210214 150153 097109 140160 238246

AO3806 , 163167 166186 286334 115118 129129 151151 214214 150153 091109 140172 218218

AO3807 , 163199 186186 326326 115124 129129 155155 202214 150153 091097 156160 214218

AO3808 , 179199 186190 298326 124133 129129 147151 202214 150150 091091 152160 218234

AO3809 , 127163 186190 286322 115133 129129 139143 202206 150150 097109 144156 230234

AO3810 , 171171 186186 310322 124145 129135 143155 218222 134153 097103 160160 218246

AO3811 , 167171 186186 314314 127145 129129 000000 000000 134150 097136 152152 186250

AO3812 , 163171 186186 322334 136142 129129 155155 226230 134153 094103 152152 242250

AO3813 , 143171 186186 326338 118121 129129 155163 206214 127153 103109 140152 238246

AO3814 , 167167 186186 294326 118136 129129 147151 202210 150153 091109 160164 218246

AO3815 , 143171 186186 310310 124142 129135 143163 218218 134150 097097 152160 186246

AO3816 , 127143 186186 322322 124136 129135 155163 222234 134153 112118 152160 186254

AO3817 , 143171 186186 322334 136136 129129 143155 190214 150153 118118 152160 230262

AO3818 , 127171 186186 290342 118124 135135 143143 190218 134150 094097 152160 186246

AO3819 , 171175 186186 290290 127130 135135 151171 206218 134153 097097 152160 186258

AO3820 , 171171 186186 290306 115121 129135 143143 226230 134150 094097 160160 246250

AO3821 , 127191 186186 310342 127127 129129 147155 202230 153153 097097 160160 238290

AO3822 , 163175 186186 290290 127130 129135 151163 218218 134153 097097 152152 186262

AO3823 , 127199 186186 298314 133136 129129 155163 214222 150150 097115 160164 234246

AO3824 , 127167 186186 294326 118136 129129 147147 202214 150153 097097 160164 246262

AO3825 , 127179 186186 274326 118133 129129 147155 206222 150150 091097 140148 206210

AO3826 , 127199 186190 298310 118148 129129 147155 202210 134150 097097 156156 206218

pop

AO2295 , 143171 186186 294322 127142 129135 155163 206210 150150 118118 152172 230250

AO2296 , 127171 186186 322342 118127 129129 151155 206214 150153 094097 152160 230234

AO2297 , 127143 186186 310322 124136 129129 151163 190222 134153 094112 152160 230238

AO2298 , 143167 186186 318322 124127 129135 151163 206218 134150 094118 152172 186266

AO2299 , 127143 166186 306306 121124 129135 155155 202206 150153 118133 140156 238250

AO2300 , 171171 186186 322326 124136 129129 155155 202230 134153 118136 152156 242242

AO2301 , 143191 186186 342346 124127 129129 155159 202206 150153 094118 152160 186242

AO2302 , 167171 186186 326318 124127 129129 151155 206214 134153 103103 140160 242254

AO2303 , 143163 186186 290326 130142 129129 143143 222234 134153 097097 140152 194242

AO2304 , 143163 186186 306326 121136 129129 155155 202230 134153 103136 156160 234286

AO2305 , 143171 166186 326342 124130 129129 143155 190234 150153 094118 160160 230238

AO2306 , 127143 186186 322346 124130 129135 155155 210234 150153 103133 156164 226266

AO2307 , 171191 186186 310342 121127 129129 155159 202214 150150 097103 160160 234242

AO2308 , 143167 186186 318346 124124 129135 143143 210214 153153 097097 156160 234250

AO2309 , 127143 186186 326326 121124 129129 147163 206230 150153 094109 152160 226266

AO2310 , 163171 186186 290326 121124 129129 143155 214214 134153 118118 140152 246246

AO2311 , 167167 186186 322322 121124 129129 151171 226226 153153 097097 152156 270270

AO2312 , 127171 186186 310318 115127 129135 155163 214218 134153 118136 152160 242246

AO2313 , 167171 186186 290314 136142 129129 151151 206210 134150 094136 160160 238250

AO2314 , 167171 186186 310322 121124 129129 151163 202214 134153 103136 140160 186246

AO2315 , 127167 186186 322326 118127 129135 151171 206222 150153 112136 160168 186238

AO2316 , 143171 186186 306342 118124 129129 147155 202210 134153 097103 156160 242258

AO2318 , 143171 186186 290322 124127 129129 151163 190206 150153 097118 152160 230266

AO2319 , 127191 186186 322322 130142 129129 155155 226230 134153 097136 152152 178242

AO2320 , 143171 186186 306334 127136 129135 143147 202230 153153 097097 160160 214242

AO2321 , 143191 186186 290322 127130 129129 151155 214222 150150 097097 160160 250254

AO2322 , 127167 186186 322334 118124 129135 151167 226226 153153 097103 156160 190226

AO2323 , 143143 166186 318346 118124 129129 155155 218230 153153 097103 152156 194246

AO2324 , 127167 186186 290322 118124 129129 151151 218222 150153 097109 156160 186254

AO2325 , 127143 186186 322322 136142 129135 143151 230230 153153 094118 152152 226286

AO2326 , 127163 186186 322322 121142 129129 155155 226234 134153 097106 152160 186258

AO2327 , 167171 166186 310314 118124 129129 151171 218226 153153 097136 156164 242250

AO2328 , 127163 186186 306318 130136 129135 155155 214230 134153 097118 160172 194230

AO2329 , 143171 186186 318322 115142 129135 143155 202230 150150 118118 152152 194238

AO2330 , 143171 186186 326326 121124 129129 155155 214214 153153 103136 152160 246246

AO2331 , 163171 186186 322334 136142 129129 155155 202234 134134 118118 152164 238242

AO2332 , 127171 186186 322326 124130 129129 151151 230234 150153 112118 160164 226226

AO2333 , 127163 166186 290342 118127 135135 163163 214230 134150 103112 160160 214226

AO2334 , 163171 186186 290334 118136 129129 143155 202230 153153 103103 140160 190194

AO2335 , 143143 186186 318322 127136 129129 155163 206210 134150 103136 160160 226250

AO2336 , 127127 186186 326342 121124 129120 151151 202222 153153 097118 160160 238238

AO2337 , 127167 186186 322342 121124 129129 155155 202222 134153 097118 140160 238238

AO2338 , 171171 186186 310326 121124 129129 155163 214226 150153 097118 152160 214258

AO2339 , 171191 186186 290322 127130 129129 147151 214214 150153 097097 152160 222242

AO2340 , 171171 186186 322322 124148 129129 155155 206214 150153 097136 152156 186214

AO2341 , 167171 186186 310322 124136 129135 143171 226226 153153 097097 152152 258270

AO2342 , 127163 186186 326342 121124 129129 143163 214230 153153 112118 160160 226246

AO2343 , 127167 186186 318322 124124 129129 155163 202210 134153 118136 152152 238250

AO2344 , 127163 186186 318342 118124 129129 155155 210214 153153 097103 152160 258262

AO4364 , 167171 166166 322322 124127 129129 155155 206230 153153 097097 152160 186194

AO4365 , 143171 166166 294334 127136 129129 155155 206234 134134 097136 160172 242266

AO4366 , 143163 186186 322322 118148 129135 155155 202226 153153 103118 156164 178242

AO4367 , 127171 186186 326342 118121 129129 151155 202214 153153 118118 160160 226234

AO4368 , 167191 186186 322322 136142 129129 155167 210226 134150 097118 152160 178250

AO4369 , 167171 166186 290314 127136 129129 155163 206206 150153 103118 160164 186230

AO4370 , 127167 166186 290318 124136 129129 143151 218234 134153 112118 160172 242242

AO4371 , 167171 186186 322322 130136 129129 143151 218226 150153 103112 152152 226246

AO4372 , 163171 186186 322326 121127 129129 147147 210230 153153 097118 152152 242250

AO4373 , 143163 186186 322334 118148 129135 151155 202218 134153 103118 156160 186242

AO4374 , 143163 186186 326338 136136 129129 143171 222230 134153 097136 152164 194250

AO4375 , 143167 186186 310326 127136 129129 143163 206230 134134 094133 160160 230242

AO4376 , 127143 186186 290322 124136 129135 151155 206222 134150 103112 152160 218254

AO4377 , 143171 186186 290322 121124 129135 151171 206214 134134 097118 140160 246254

AO4378 , 167171 186186 322322 121136 129129 151167 218230 153153 097097 152156 186242

AO4379 , 163171 186186 318326 121142 129129 151163 206234 153153 103136 160160 186270

AO4380 , 127143 186186 310322 124142 129135 155167 206230 153153 094118 152164 226242

AO4381 , 143171 186186 310322 127136 129129 151163 218230 134153 103118 152160 186250

AO4382 , 127171 186186 290322 121142 129129 155163 190214 134153 103112 152160 230246

AO4383 , 167171 186186 322346 124124 129129 151155 214218 153153 097097 152156 238246

AO4384 , 127143 186186 322334 127127 129129 155163 202218 134134 094118 152164 186238

AO4385 , 143143 166186 322322 136136 129129 147155 214218 150153 094112 152152 238238

AO4386 , 171171 186186 322326 121136 129129 151155 214214 134153 097118 160160 238246

AO4387 , 143171 186186 322326 121148 129129 155155 206206 134153 097097 152168 186242

AO4388 , 143171 186186 318342 136142 129135 155155 210214 150153 112136 160160 242250

AO4389 , 143171 186186 322322 121127 129129 155163 214234 150153 103118 152152 186262

AO4390 , 167171 186186 290314 127127 129129 155163 206210 150153 097103 160164 186250

AO4391 , 167171 166186 290322 124124 129135 143147 218234 134153 097097 160164 242242

AO4392 , 143171 186186 310322 127127 129129 155167 206214 153153 094103 160164 226270

AO4393 , 127127 186186 290310 118124 129135 151155 206206 134153 118118 152160 190270

AO4394 , 167167 186186 310318 121124 129129 143151 214218 153153 097097 160160 234246

AO4395 , 143167 186186 322342 124124 129129 143155 222222 150153 118118 160168 250254

AO4396 , 143167 166186 322326 121142 129135 155155 210214 134153 097112 152152 246250

AO4397 , 143143 186186 318322 121124 129129 151171 206218 134153 097136 156160 186218

AO4398 , 167171 166186 322322 136136 129129 151151 190214 150153 097118 152160 230246

AO4399 , 127171 186186 290322 127136 129129 151155 214230 134134 097097 140160 226230

AO4400 , 127163 186186 322334 124124 129129 147155 214214 134153 097103 156164 246258

AO4401 , 143171 186186 310338 124136 129135 143155 226234 150153 097097 152152 238258

AO4402 , 167171 166186 322326 121127 129129 151155 218226 150153 118118 152160 242250

AO4403 , 127143 186186 318318 124136 129129 155155 202206 150153 118118 152152 238266
